# Author Correction: High voltage electrolytes for lithium-ion batteries with micro-sized silicon anodes

**DOI:** 10.1038/s41467-024-47037-6

**Published:** 2024-03-26

**Authors:** Ai-Min Li, Zeyi Wang, Travis P. Pollard, Weiran Zhang, Sha Tan, Tianyu Li, Chamithri Jayawardana, Sz-Chian Liou, Jiancun Rao, Brett L. Lucht, Enyuan Hu, Xiao-Qing Yang, Oleg Borodin, Chunsheng Wang

**Affiliations:** 1 Department of Chemical and Biomolecular Engineering, University of Maryland, College Park, MD 20740 USA; 2 Battery Science Branch, DEVCOM Army Research Laboratory, Adelphi, 20783 MD USA; 3 Chemistry Division, Brookhaven National Laboratory, Upton, NY 11973 USA; 4 Department of Chemistry and Biochemistry, University of Maryland, College Park, MD 20740 USA; 5 Department of Chemistry, University of Rhode Island, Kingston, RI 02881 USA; 6 Maryland Nanocenter, University of Maryland, College Park, MD 20740 USA

**Keywords:** Batteries, Batteries, Energy

Correction to: *Nature Communications* 10.1038/s41467-024-45374-0, published online 08 February 2024

In this article, the *y*-axis labels for Fig. 8b, d and Supplementary Figs. 30, 31, and 32b were wrongly labeled as “Potential (V vs Li/Li^+^)” but should have been “Cell voltage (V)”; the figures should have appeared as shown below. Besides, the caption for Fig. 4b–d was incorrectly given as “Electron localized function and *E*_int_ between the Li_x_Si (Li_15_Si_4_, Li_12_Si_7_, and LiSi) alloys and major SEI components (LiF, Li_2_O and Li_2_CO_3_), the color differences indicate the *E*_int_ value (from 0.0 to 1.0) with the scale bar shown on the left” but should have been “Electron localized function and *E*_int_ between the Li_x_Si (Li_15_Si_4_, Li_12_Si_7_, and LiSi) alloys and major SEI components (LiF, Li_2_O and Li_2_CO_3_) with the scale bar (from 0.0 to 1.0) shown on the left.”

The original article has been corrected.

### Supplementary information

Updated Supplementary Information

