# Supplementary material for: High voltage electrolytes for lithium-ion batteries with micro-sized silicon anodes
Source: Nat Commun. 2024 Feb 8;15:1206. doi: 10.1038/s41467-024-45374-0 (PMC10853533; doi:10.1038/s41467-024-45374-0)
Supplement: Supplementary file 1 — Supplementary Information [file 41467_2024_45374_MOESM1_ESM.docx]

**High Voltage Electrolytes for Lithium-Ion Batteries with Micro-Sized Silicon Anodes**

Ai-Min Li^1^, Zeyi Wang^1^, Travis P. Pollard^2^, Weiran Zhang^1^, Sha Tan^3^, Tianyu Li^4^, Chamithri Jayawardana^5^, Sz-Chian Liou^6^, Jiancun Rao^6^, Brett L. Lucht^5^, Enyuan Hu^3^, Xiao-Qing Yang^3^, Oleg Borodin^2*^ and Chunsheng Wang^1*^

^1^Department of Chemical and Biomolecular Engineering, University of Maryland, College Park, Maryland 20740, United States

^2^Battery Science Branch, DEVCOM Army Research Laboratory, Adelphi, 20783, Maryland, United States

^3^Chemistry Division, Brookhaven National Laboratory, Upton, NY, United States

^4^Department of Chemistry and Biochemistry, University of Maryland, College Park, Maryland 20740, United States

^5^Department of Chemistry University of Rhode Island South Kingstown, RI 02881, USA

^6^Maryland Nanocenter, University of Maryland College Park, MD 20740 (USA)

E-mail: [cswang@umd.edu](mailto:cswang@umd.edu); oleg.a.borodin.civ@army.mil

**Table of Contents:**

1. Supplementary Tables 1*–*2

2. Supplementary Notes 1*–*6

3. Supplementary Figures 1*–*35

4. Supplementary References

***1. Supplementary Tables 1****–****2***

Supplementary Table 1. Comparison of the state-of-the-art battery performances using micro-sized silicon as anode.

| **Electrolyte** | **µSi Size (µm)** | **Pretreatments** | **Loading**  **(mAh cm^-2^)** | **Cathodes (N/P ratio)** | **Voltage Range (V)** | **Cycle Capacity**  **(mAh.g^-1^)** | **Cyclability** | **Ref** |
| --- | --- | --- | --- | --- | --- | --- | --- | --- |
| LP40 | 4.6 | PFM Binder | ~0.67 | none | 0.01V*–*1V | 3200 (0.04C)  /2500 (0.5C) | Li\|\|μSi: No C-rate  0%-5 cycles | [**1**](#_ENREF_1) |
| LP40 | 4.6 µSi + nano Si | PFM Binder | ~0.97 | none | 0.01V*–*1V | 3200 (0.04C)  /2500 (0.5C) | Li\|\|μSi: No C-rate  75%-30 Cycles | [**1**](#_ENREF_1) |
| LP40+ 10% FEC + 1 % VC | 1*–*3 | Encapsulated in graphene cage | ~3.0 | LCO  (~1.13) | 0.01V*–*1V;  3V*–*4.2V | 3300 (0.05C)  /1600 (0.5C) | μSi\|\|LCO: ~1/3C  90%-100 Cycles | [**2**](#_ENREF_2) |
| LP40 + 7.5% FEC + 0.5% VC | ~2.1 | PR-PAA binder | ~3.3 | NCA  (~1.15) | 0.01V*–*1.5V;  2.7V*–*4.3V | 2971 (0.033C)  2600 (0.2C) | μSi\|\|NCA: 0.2C  98%-50 cycles | [**3**](#_ENREF_3) |
| 1.0 M LiPF_6_-EC/DMC (1:1) + 10% FEC | 2*–*6 | HEA-co-DMA binder; 0.5-3 µm | ~1.93 | NMC111  (~1.1) | 0.01V*–*1.2V;  2.8V*–*4.2V | 2850 (0.1C)  /2394 (0.25C) | μSi\|\|NMC111: 0.2C  80.8%-120 cycles | [**4**](#_ENREF_4) |
| LP40 + 4% FEC | 3*–*8 | self-healing conductive polymer | 1.5*–*2.1 | none | 0.01V*–*1V | 2617 (0.1C)  /2500 (0.1C) | Li\|\|μSi:~1/10  80%-90 cycles | [**5**](#_ENREF_5) |
| 2.0 M LiPF_6_-MixedTHF | 1*–*5 | none | ~2.5 | LFP  (~0.77)  NCA  (~0.77) | 0.06V*–*1V;  LFP:  2.5V*–*3.45V;  NCA:  2.7V*–*4.1V | 3200 (0.1C)  /2800 (0.2C) | μSi\|\|LFP: 0.3C  80%-100 cycles;  Li\|\|NCA: 0.3C  92%-30cycles | [**6**](#_ENREF_6) |
| **FST** | **1***–***5** | **None** | **~2 and ~4** | **NCA**  **(~1.1)** | **0.05V***–***1V;**  **NCA:**  **2.7V***–***4.3V** | **3380 (0.05C)**  **>2700 (0.25C)** | **Li**\|\|**μSi:**  **0.25C 80%-250 cycles**  **μSi**\|\|**NCA coin cell: 0.2C,**  **81%-200 cycles;**  **100 mAh pouch cell:**  **0.2C, 89%-120 cycles** | **This work** |

Note: LP40: 1.0 M LiPF_6_-EC/DEC (1:1 by weight)

Supplementary Table 2. FST electrolytes properties predicted from MD simulations.

| Temperature | 90 °C | 60 °C | 25 °C |
| --- | --- | --- | --- |
| cumulative equilibration runs (ns) | 29 | 61.9 | 39.8 |
| cumulative production runs (ns) | 166 | 173.7 | 147 |
| Diffusion coefficients (10^-10^ m^2^ s^-1^) | | | |
| SL | 4.61 | 2.4 | 0.89 |
| FEC | 5.96 | 3.3 | 1.26 |
| TTE | 5.04 | 2.9 | 1.05 |
| PF_6_^-^ | 4.33 | 2.3 | 0.89 |
| Li^+^ | 3.67 | 1.9 | 0.74 |
| Conductivity (mS cm^-1^) | 13.6 | 8.2 | 4.2 |
| viscosity (mPa * s) | 3.1 | 5.7 | 16.8 |
| degree of uncorrelated ion motion (ionicity) | 0.60 | 0.6 | 0.68 |
| Li^+^ transference number (t_+_) | | | |
| α (Roling) | 0.44 | 0.50 | 0.45 |
| β (Roling) | 0.40 | 0.35 | 0.30 |
| $t_{+}^{abc}$ | 0.61 | 0.67 | 0.59 |
| Li^+^ cation coordination number | | | |
| O (TMS) within 2.8 Å | 2.74 | 2.69 | 2.91 |
| O_c_ (FEC) within 2.8 Å | 0.66 | 0.84 | 0.78 |
| F (PF_6_) within 2.8 Å | 0.93 | 0.84 | 0.73 |
| P (PF_6_) within 4.4 Å | 0.79 | 0.73 | 0.65 |
| Fraction of free anions, no counterion within r(Li-P) of 4.4 Å | | | |
| fraction of PF_6_^-^ not bound to Li^+^ | 0.39 | 0.41 | 0.46 |
| fraction of Li^+^ not bound to PF_6_^-^ | 0.36 | 0.39 | 0.45 |

***2. Supplementary Notes 1****–****6***

**Supplementary Note 1**

*Non-flammability test of the proposed electrolyte*

The SL molecule has been recognized as one of the flame retardants along with other phosphorus-centered regents.[^7^](#_ENREF_7)^,^[^8^](#_ENREF_8) All fluorinated electrolyte is clarified as non-flammable electrolytes. Herein, besides the unique electrochemical benefits contributed by this optimized FST formulation, one additional advantage is its non-flammability, which is highly desirable but often unavailable from other non-aqueous electrolytes. As demonstrated in flaming tests (Supplementary Fig. 6, Supplementary videos 1*–*3 for EE, FFT, and FST electrolytes, respectively), this FST electrolyte does not burn following ignition, in sharp contrast with the highly flammable EE electrolyte. This is attributed to two facts: one is the fluorination on FEC, TTE molecules, which can effectively serve as an inhibitor to the propagation of oxygen radicals during combustion;[^9^](#_ENREF_9)^,^[^10^](#_ENREF_10) second is the SL itself can act as flame retardant.[^7^](#_ENREF_7)^,^[^8^](#_ENREF_8)

**Supplementary Note 2**

*Discussion on the solubility of organic SEI in the studied electrolytes.*

The well-established and identified organic component in the SEI, LiEMC[^11^](#_ENREF_11)^,^[^12^](#_ENREF_12), is used as a case example to verify the solubility of the organic components in the proposed electrolytes. The LiEMC standard sample is chemically synthesized based on our reported method.[^11^](#_ENREF_11) As shown in Supplementary Fig. 8, to the freshly prepared ~ 0.5 mL EE, FFT, and FST electrolytes, ~ 10 mg of LiEMC was added as a fine powder. Then the vials were sealed with electric tape and thoroughly mixed with a mixer for two hours. All three vials showed some un-dissolved particles on the glass wall, indicating the difficulty of re-dissolving LiEMC back into the mother electrolytes where it has been formed. After a careful filtration of the electrolytes inside a glovebox, the ^1^H-NMR spectra were collected for all three samples for a detailed comparison. A co-axis NMR technique is applied with external C_6_D_6_ as a reference for the in-situ ^1^H-NMR measurement. Supplementary Fig. 9a shows the full ^1^H-NMR spectra of the three electrolytes together with the LiEMC standard dissolved in *d^6^*-DMSO. Note that the LiEMC itself undergoes an equilibrium reaction that happens in the *d^6^*-DMSO solvents,[^11^](#_ENREF_11) such that only the signature LiEMC peaks at 3.67 ppm and 3.42 ppm were chosen for a clear comparison. Supplementary Fig. 9b shows an enlarged view between 3.8 ppm to 3.4 ppm for all samples, with the electrolytes signal intensity magnified by 100 times. The EE electrolyte shows ambiguous signals for the LiEMC, while no obvious signal was found for the FFT electrolyte, even after the 100 times magnification, which makes sense as the fluorinated of the carbonate electrolytes will reduce its solvation ability, such limits the solubility of organic species. On the other hand, for the FST electrolytes, an apparent signal could be identified as the LiEMC, though the signal somehow low-field shifted in the FST electrolytes compared to the *d^6^*-DMSO standard. Nevertheless, the result here clearly shows that the strong solvation ability of sulfolane molecules could indeed dissolve some of the organic components in the SEI. As such, only the insoluble inorganic species like LiF and Li_2_O will be accumulated in the SEI, which will guarantee a ceramic LiF-Li_2_O SEI that is designed for the success cycle of SiMPs.

**Supplementary Note 3**

*Discussion of the SEI structure from different electrolytes*

There are contrasting composition differences among the SEI formed in the three electrolytes, EE, FFT, and FST, with the outstanding ones being the C- and O-content as well as their trends during the Ar^+^ sputtering, as illustrated in the main text. Supplementary Fig. 13 shows the atomic ratio of all elements found in the SEI layer cycled in different electrolytes. Obvious differences were observed for the C, O, and F signals: for μSi electrode cycled in EE and FFT electrolytes, the atomic composition remained the same from the surface to the inner part till 600 S sputtering, indicating an evenly distributed SEI with all species mixed, that is, the formation of organic and inorganic species happened at the same time with no preference in these two reference electrolytes. The high content of C- and O- signals showed up at the same time in EE electrolytes implies organic fragment formation such as ROLi, LiOCOR, or Li_2_CO_3_, all of which show poor resistance of the large volume expansion during the cycling of the μSi anode. Though the F-content has been largely improved in the FFT electrolytes, the high ratio and evenly accompanying C-content signifies the organic-dominated SEI, with less amount of LiF embedded inside the organic matrix, resulting in a less uniform and less compact SEI. This causes more electrolyte penetration through the SEI and further electrolyte decomposition on the SiMPs surface, forming a thicker SEI, as proved by the thickness evolution test during cycling (Supplementary Fig. 24), all of which leads to increasing impedance in the EIS spectra (Supplementary Fig. 17b), low cycling CE (<99%) and quick capacity fading. For the designed FST electrolytes, the O-ratio showed a clear increase from the surface to the inner part of the SEI during the sputtering, along with a C-ratio decrease, indicating enrichment of inorganic Li_2_O, and a large decrease of organic species. The more Li_2_O formation along with LiF in the SEI can be attributed to two factors: 1) more SL solvent decomposition due to the much stronger solvation ability of SL compared to FEC or TTE, as confirmed by Raman spectra (Supplementary Fig. 2a) and MD simulations (Supplementary Figs. 2, 4). 2) the initial reduction of FEC helps to form the LiF-rich inner core, which favors the following reduction of SL to form Li_2_O. Two weak S*2p* signals at 170 eV and 163 eV were also observed in the FST electrolytes (Supplementary Fig. 12), which can be ascribed to Li_2_SO_3/4_ and Li_2_S, indicating the complete reduction of the SL molecule, leaving limited organic species in the SEI.

**Supplementary Note 4**

*Discussion of electron localized function for the Li_x_Si alloy–SEI components interfaces*

In the Li*_x_*Si region, the red basin with an ELF value of 0.8*–*1 was observed around the Si core while a flat green profile with an ELF value of around 0.3*–*0.5 dispersed (Fig. 4b*–*d, main text). With the increase of Si content (from Li_15_Si_4_ to Li_12_Si_7_ then to LiSi), the ELF value of the red basin increases, representing more covalent characteristics between Si-Si bonds. Meanwhile, the flat green region shrinks when Li content decreases due to fewer metallic bonds in the Li*_x_*Si phase. ELF map in LiF and Li_2_O region shows a sphere shape with an ELF value of ~0.8, representing ionic bonds with charge completely transferred between ions. The bond basins were observed within the Li_2_CO_3_ region due to the covalency of C-O bonds. In the boundary of Li*_x_*Si and Li*_y_*X, a region with an ELF value of <0.2 was observed for LiF|Li*_x_*Si, Li_2_O|Li*_x_*Si interfaces, indicating the absence of bonding between the atoms at the interface. In contrast, the ELF value between the Li_2_CO_3_|Li*_x_*Si interfaces varies from 0 to 0.9, corresponding to the formation of mixed ionic and covalent bonds. Therefore, the interfacial energy for Li_2_O|Li*_x_*Si interfaces is comparable to LiF|Li*_x_*Si and is much higher than that of Li_2_CO_3_|Li*_x_*Si interfaces, validating the Si-phobic of Li_2_O with weak bonding to Li_x_Si, which will release the SEI stress during the volume change of SiMPs and improve the μSi anode cycle performance.

**Supplementary Note 5**

*Discussion of Li_2_O/LiF composite SEI on improving lithium-ion transport*

The ionic conductivity of Li_2_O/LiF composite SEI was found to be much higher than pure Li_2_O or LiF SEI due to the space charge effect.[^13^](#_ENREF_18) The formation of space charge region in Li_2_O-LiF interface can be attributed to different types of ionic carrier and diffusion mechanisms in Li_2_O and LiF (Supplementary Fig. 15). More specifically, the dominant diffusion carrier in Li_2_O is Li-ion interstitial, while in LiF is Li-ion vacancy from Schottky defects owing to lower formation energy. Therefore, lattice Li-ion in LiF will spontaneously migrate towards Li_2_O lattice to form interstitial defect as evidenced by the negative Gibbs free energy of the defect reaction in the Li_2_O-LiF interface (see Method part below for more details). In this model, the topological distribution of the LiF and Li_2_O phases was simplified as alternatively parallel so that the Li^+^ conduction path could penetrate along the SEI (Supplementary Fig. 16a). The simplified model provides an upper limit estimation of the ionic conductivity in actual SEI, where the tortuosity factor also affects Li^+^ conduction significantly. The defect reaction was found to boost the interstitial Li^+^ defect concentration in the Li_2_O lattice near the LiF-Li_2_O interface up to 10^4^ times and reduce the electron concentration to 10^-4^ compared with that of bulk Li_2_O. (Fig. 6e, main text) According to the space charge model, when only a 5% volumetric percentage of LiF was added to Li_2_O with a grain size of 15 nm, the ionic conductivity of SEI increased from 3.0*10^-5^ mS cm^-1^ in pure Li_2_O to 2.0*10^-3^ mS cm_-1_ in Li_2_O-LiF composite. (Fig. 6f, main text) Further reducing the grain size of Li_2_O and increasing the amount of LiF can generate more Li_2_O-LiF interface and improve the contribution of space charge effect to total conductivity. In this work, the grain size of Li_2_O was estimated to be < 3nm based on TEM observation (Supplementary Fig. 14) and the volumetric percentage of LiF is about 18% from XPS spectra (Supplementary Fig. 13, more details in Method part 3 below). Therefore, the total ionic conductivity of Li_2_O-LiF composite SEI was predicted to be ~2.5*10^-2^ mS cm^-1^.

**Methods**

*1. Identifying the dominant point defect of LiF and Li_2_O*

Dominant Li point defect of LiF and Li_2_O at the Li anode was identified based on defect formation energy calculation. The formation energy of point defect X with charge q is (1):


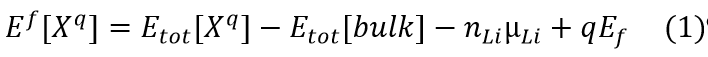


Where $E_{tot}[X^{q}]$ and $E_{tot}[bulk]$ are the DFT total energy of LiF or Li_2_O supercell with and without defect. $\boldsymbol{n}_{\boldsymbol{Li}}$ is the number of Li atoms (ions) added to or removed from the supercell. $\boldsymbol{\mu}_{\boldsymbol{Li}}$ is the chemical potential of Li. $\boldsymbol{E}_{\boldsymbol{f}}$ is Fermi level referenced to the valence band minimum (VBM). By requiring overall charge neutrality, the Fermi level can be determined from equation (2)


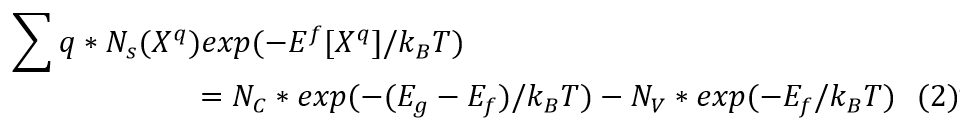


Where $\boldsymbol{N}_{\boldsymbol{s}}\boldsymbol{(}\boldsymbol{X}^{\boldsymbol{q}}\boldsymbol{)}$ is the number of sites and defects $\boldsymbol{X}$ can be generated per unit volume. $\boldsymbol{N}_{\boldsymbol{C}}$ and $\boldsymbol{N}_{\boldsymbol{V}}$ are the integrated density of states (DOS) of the conduction and valence band. $\boldsymbol{E}_{\boldsymbol{g}}$ is the bandgap of LiF or Li_2_O bulk supercell. Temperature $T$ is 300 K. $\boldsymbol{k}_{\boldsymbol{B}}$ is Boltzmann’s constant. The calculations were performed with the help of Pymatgen[^13^](#_ENREF_13) and Pycdt[^14^](#_ENREF_14) software.

*2. Defect distribution and ionic conductivity in space charge region*

The space charge model was set up as previously reported.[^13^](#_ENREF_18) Possible Li point defects reactions in the interface of LiF and Li_2_O were listed as follows:


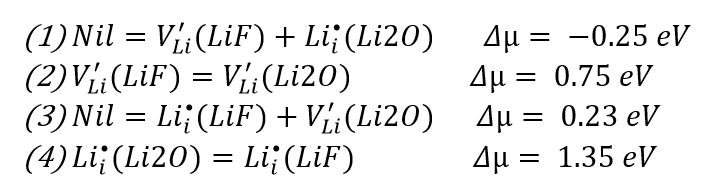


Here, $\boldsymbol{\Delta\mu}$ is the reaction free-energy of point defects and was calculated based on defect formation energy from DFT calculations and the reported data of published papers by the same author for consistency.[^15^](#_ENREF_15) We can see, only reaction (1) has a negative chemical potential difference, which can occur spontaneously. That is, when Li_2_O is in contact with LiF, Li-ion will leave the LiF lattice and accumulate in the Li_2_O lattice as interstitial defects. The concentration of interstitial Li^+^ defect and electron in Li_2_O lattice near the Li_2_O-LiF interface can be described by equation (3):


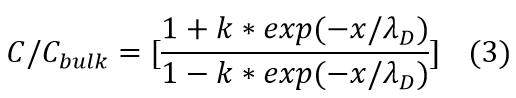


Where $\boldsymbol{C}_{\boldsymbol{bulk}}$ is the concentration of Li^+^ interstitial defect in bulk Li_2_O material. Parameter **k** is a constant and is equal to $\frac{1- {[C_{Li}^{x=0}/C_{bulk}]}^{0.5}}{1+ {[C_{Li}^{x=0}/C_{bulk}]}^{0.5}}$. Here, $C_{Li}^{x=0}$ was determined by ${C_{Li}^{x=0}=N}_{s}({Li}_{i}^{⦁})exp\{-(E^{f}[{Li}_{i}^{⦁}]+\Delta\mu)/k_{B}T\}$. $\boldsymbol{\lambda}_{\boldsymbol{D}}$ is Debye length which defined as $\lambda_{D}={(\frac{\varepsilon\varepsilon_{r}RT}{2*F^{2}C_{bulk}})}^{0.5}$. $\boldsymbol{\varepsilon}$ and $\boldsymbol{\varepsilon}_{\boldsymbol{r}}$ are vacuum and relative permittivity, respectively. $\boldsymbol{R}$ is the ideal gas constant. $\boldsymbol{F}$ is the Faraday constant. Plug in all parameters and we get the Debye length of Li_2_O is 2 nm. The normalized concentration profile of interstitial Li^+^ and mobile electron Li_2_O within the Li_2_O-LiF space charge region is shown in Fig. 6e (main text). The total ionic conductivity of the LiF-Li_2_O composite SEI as a function of the volume fraction of LiF was calculated based on Equation (4)[^16^](#_ENREF_16):


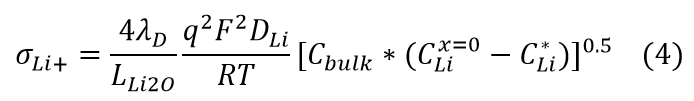


Where $\boldsymbol{L}_{\boldsymbol{Li}\boldsymbol{2}\boldsymbol{O}}$ is the size of the Li_2_O grain. $\boldsymbol{C}_{\boldsymbol{Li}}^{\boldsymbol{*}}$ is the Li^+^ interstitial defect concentration in the center of the Li_2_O grain, which can be obtained from Equation 3. $\boldsymbol{D}_{\boldsymbol{Li}}$ is calculated from Equation (5):[^17^](#_ENREF_22)


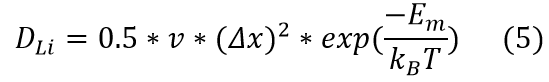


Here, $\boldsymbol{v}$ is the lattice vibration frequency (=10^13^ Hz). $\boldsymbol{\Delta x}$ is the hopping distance of interstitial Li (= 4.65 Å). $\boldsymbol{E}_{\boldsymbol{m}}$ is the migration barrier of Li^+^ in Li_2_O.[^17^](#_ENREF_17)

*3. Volumetric ratio of LiF and Li_2_O in composite SEI*

In LiF-Li_2_O composite SEI, the volumetric ratio of X (X = LiF or Li_2_O) in SEI can be calculated by equation (6):

$\vee_{X}=\nu_{m*\eta_{X}}^{X}$ (6)

The molar volume $\boldsymbol{v}_{\boldsymbol{m}}^{\boldsymbol{X}}$ can be calculated by $v_{m}^{X}=\frac{M^{X}}{\rho^{X}}$, where $\boldsymbol{M}^{\boldsymbol{X}}$ is the molar mass (LiF is 25.93 g mol^-1^ and Li_2_O is 29.88 g mol^-1^). $\boldsymbol{\rho}$ is the volumetric density (LiF is 2.635 g cm^-3^ and Li_2_O is 2.013 g cm^-3^). $\boldsymbol{n}_{\boldsymbol{X}}$ is the molar ratio of LiF and Li_2_O. Based on XPS, they are $n_{LiF}: n_{Li2O}$~ 1 : 3. Plug in all numbers we get $V_{LiF}=18\%$.

**Supplementary Note 6**

*The versatility of the electrolyte-enhanced SiO anode performance in Li||SiO half-cell*

To further demonstrate the superiority of our designed electrolyte and confirm the effectiveness of Li_2_O-dominated SEI as we proposed in the design concept, we tested the SiO anode with a high areal mass loading of ∼2.8 mg cm^-2^, corresponding to a high areal capacity of ∼4.5 mAh cm^-2^. As shown in Supplementary Fig. 17, the SiO||Li half-cell with our designed FST electrolytes retained 68% capacity after 200 cycles at 0.2C with high cycle CE >99.9 %, whereas only 17% and 32% capacity remained after 40, 100 cycles using the reference EE and FFT electrolytes, respectively, with cycle CE <98.3%. (Supplementary Figs. 18, 19). The distinct performance differences in SiMPs and SiO electrodes demonstrate the versatility of our designed FST electrolytes.

***3. Supplementary Figures 1****–****35***


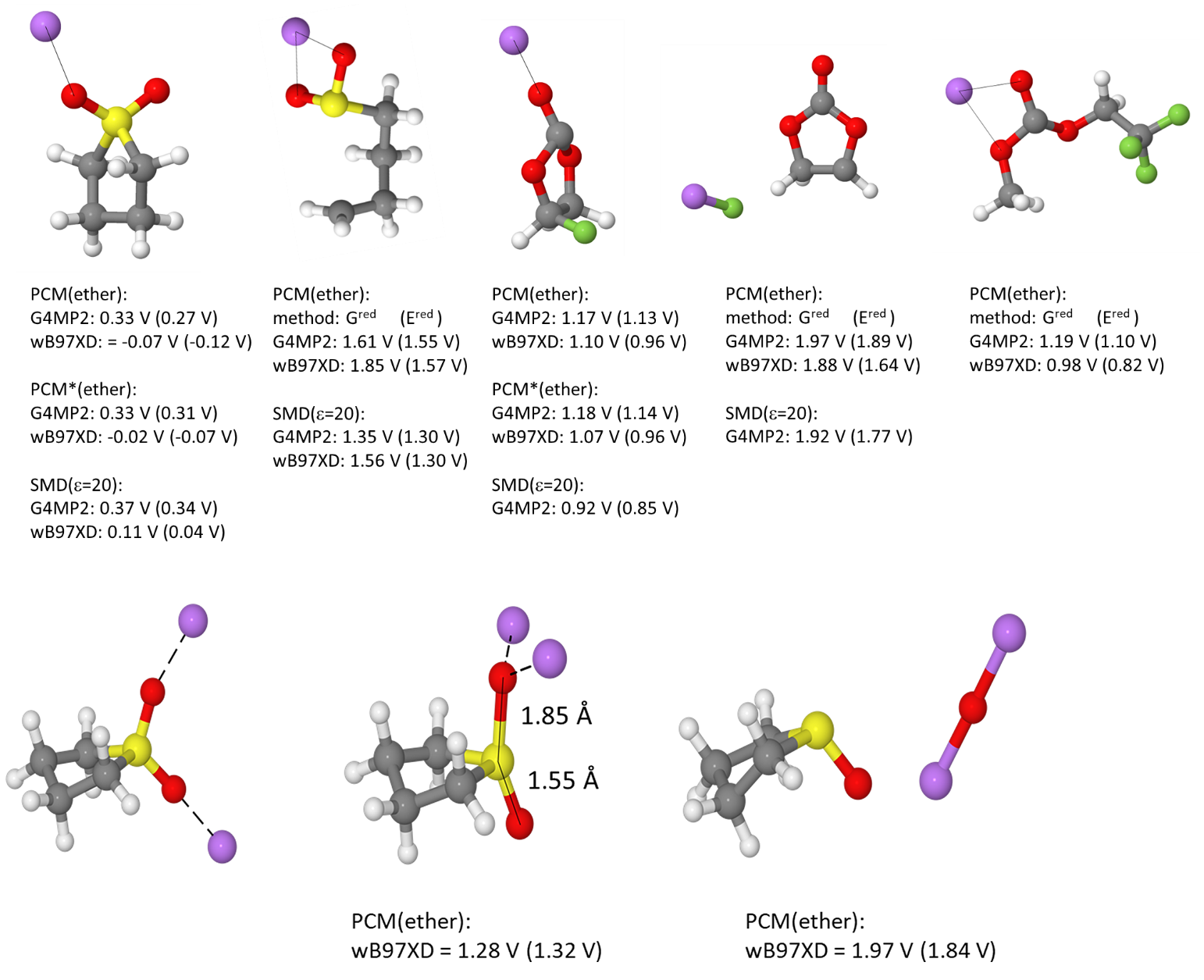


**Supplementary Figure 1**. Reduction potentials from QC calculations using G4MP2 composite methodology and ωB97XD/6-31+G(d,p) DFT calculations with all solvates immersed in implicit solvent modeled using PCM (ether) or SMD (ε = 20). The molecules are shown in the ball-and-stick model with different colors representing different elements, grey (carbon), white (hydrogen), red (oxygen), yellow (sulfur), green (fluorine) and purple (lithium). The black lines (solid and dashed) correspond to the close intact in the selected solvates.


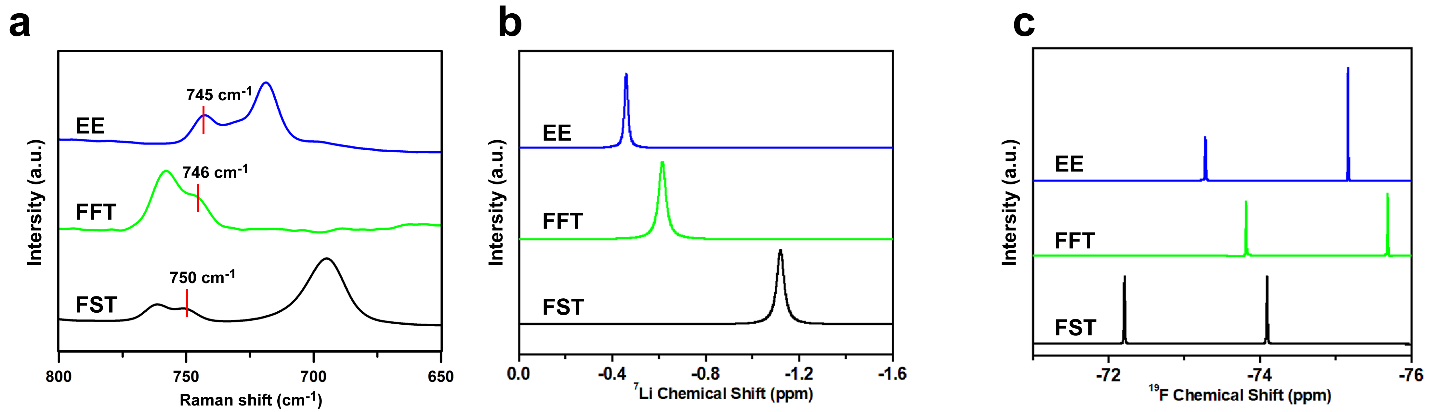


**Supplementary Figure 2.** Electrolytes solvation of 1.0 M LiPF_6_/EC-EMC (EE), 1.0 M LiPF_6_ in FEC-FEMC-TTE (FFT), and 1.0 M LiPF_6_ in FEC-SL-TTE (FST). **a**, Raman spectra in the range of 800*–*650 cm^-1^. **b***–***c,** in-situ multi-nuclear NMR spectra, all data were collected at room temperature. NMR tuning and shimming using external reference deuterated solvent of D_2_O in a co-axis NMR tube. **b**, ^7^Li-NMR. **c**, ^19^F-NMR. Source data are provided as a Source Data file.


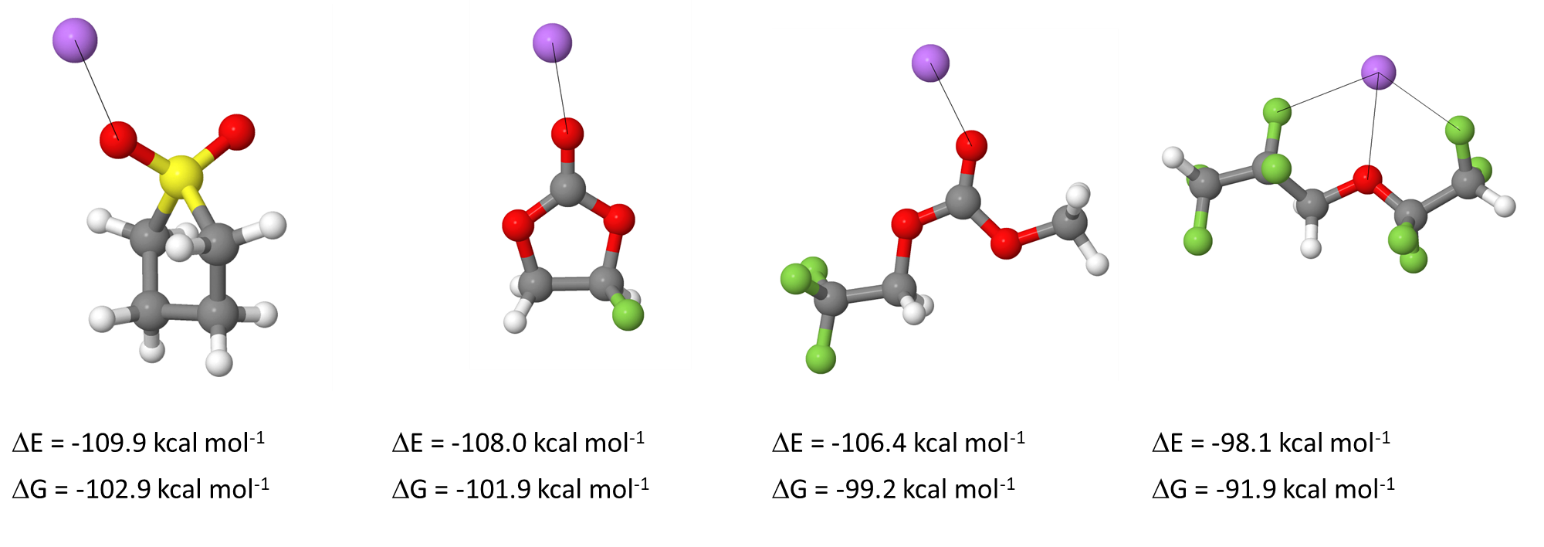


**Supplementary Figure 3.** Binding energies of Li^+^ to solvents from wB97XD/6-31+G (d, p) DFT calculations with all solvates immersed in implicit solvent modeled using PCM (ether) model. Binding energy is calculated relative to the isolated solvent in PCM (ether) and Li^+^ in the gas phase. The molecules are shown in the ball-and-stick model with different colors representing different elements, grey (carbon), white (hydrogen), red (oxygen), yellow (sulfur), green (fluorine) and purple (lithium). The black lines correspond to the close intact in the selected solvates.


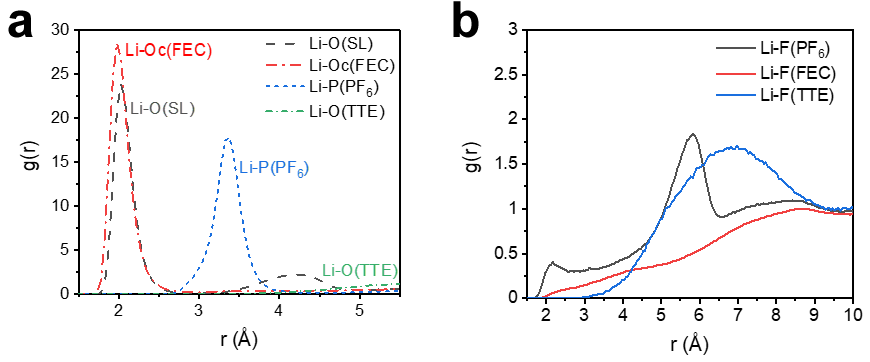


**Supplementary Figure 4**. Radial distribution functions g(r) for FST electrolyte from MD simulations at 25 °C. **a**, Li-O and Li-P interactions, **b**, Li-F interactions. Despite a slight magnitude of the higher peak for Li-Oc(FEC) g(r) compared to Li-O(SL), a significantly higher Li-O(SL) coordination number of 2.9 was observed vs 0.8 Oc(FEC) coordination number. It is due to the higher number density of SL vs FEC (630 SL vs 280 FEC in the MD simulation box) and two oxygens being present in the -SO_2_ group of SL vs only one carbonyl Oc is present in FEC.


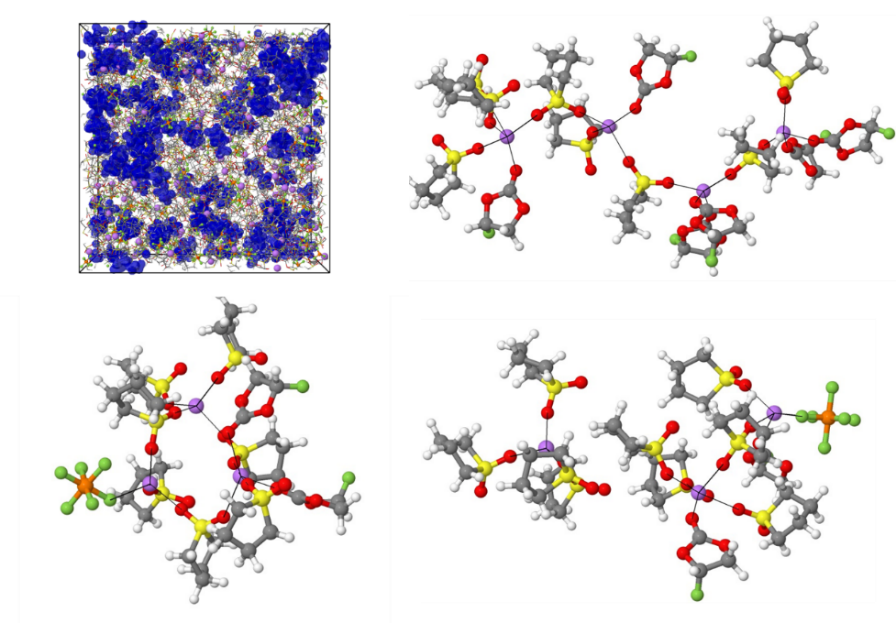


**Supplementary Figure 5**. A snapshot of MD simulation box with solvent shown as wireframes and TTE diluent highlighted using blue iso-surface. Other molecules are shown in the ball-and-stick model with different colors representing different elements, grey (carbon), white (hydrogen), red (oxygen), yellow (sulfur), green (fluorine), brown (phosphorus) and purple (lithium). The black lines correspond to the close intact in the selected solvates.


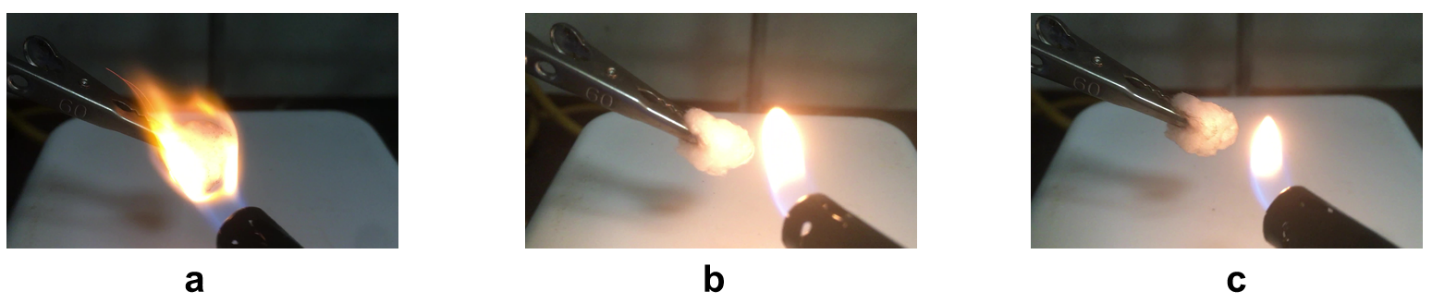


**Supplementary Figure 6**. Flammability test for the three different electrolytes: EE (**a**), FFT (**b**), and FST (**c**). These images are extracted from the supplementary videos **1** (EE), **2** (FFT), and **3** (FST).


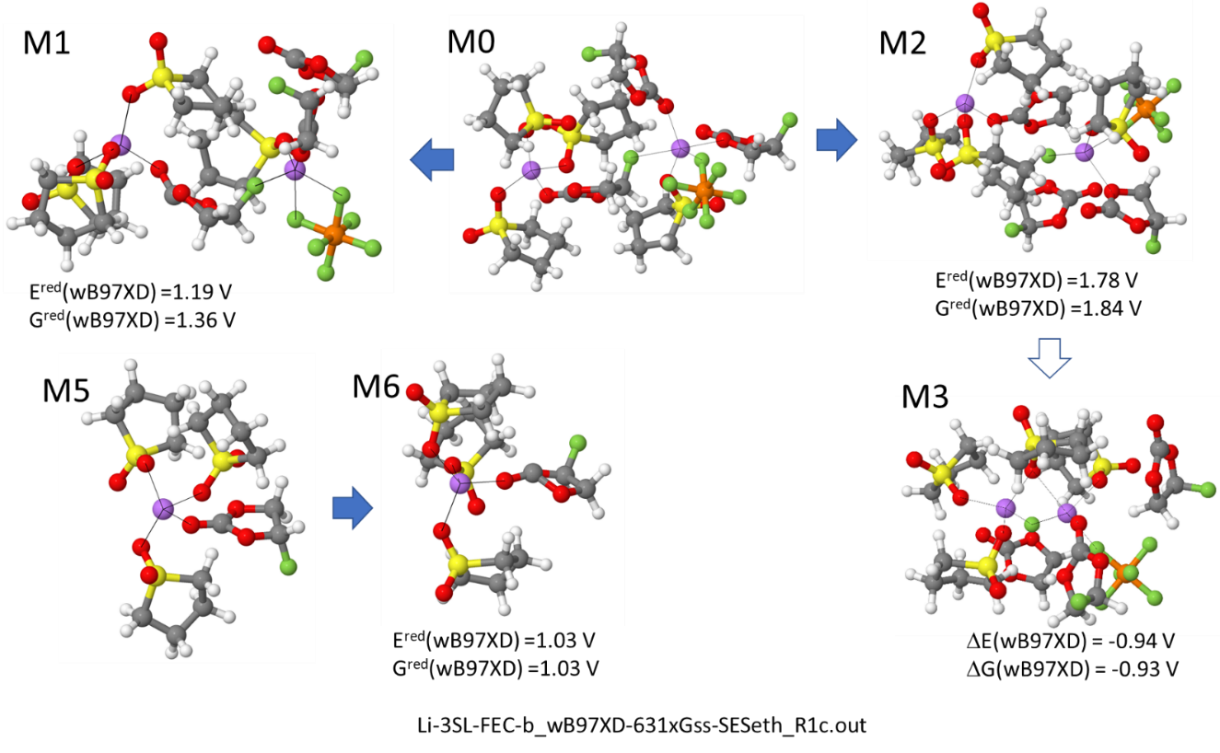


**Supplementary Figure 7**. Reduction potentials FEC from wB97XD/6-31+G(d,p) DFT calculations with all solvates immersed in implicit solvent modeled using PCM (ether) model. The initial configurations of the representative solvates were extracted from MD simulations. The molecules are shown in the ball-and-stick model with different colors representing different elements, grey (carbon), white (hydrogen), red (oxygen), yellow (sulfur), green (fluorine) and purple (lithium). The blue arrows indicate the reduction pathway, and the black lines correspond to the close intact in the selected solvates.


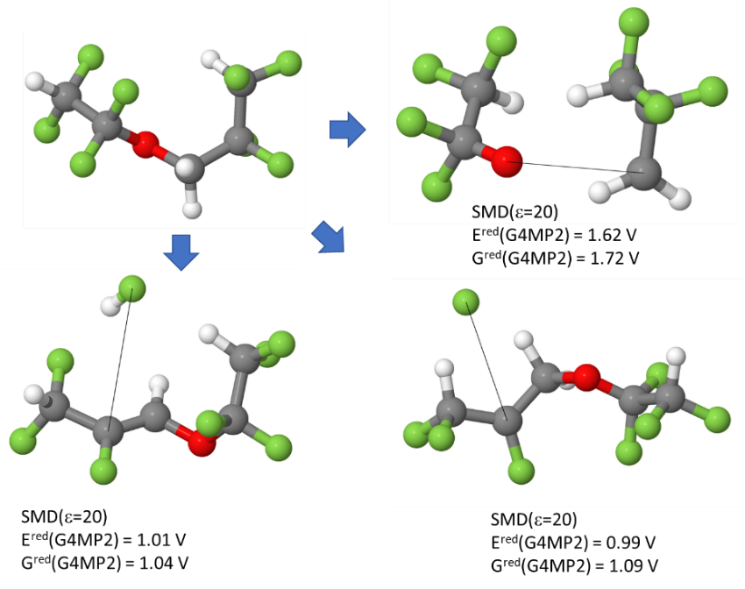


**Supplementary Figure 8**. Reduction potentials from QC calculations using G4MP2 composite methodology TTE immersed in implicit solvent modeled using SMD (ε=20) model. The molecules are shown in the ball-and-stick model with different colors representing different elements, grey (carbon), white (hydrogen), red (oxygen) and green (fluorine). The blue arrows indicate the reduction pathway, and the black lines correspond to the bond breaking in the TTE molecule.

**
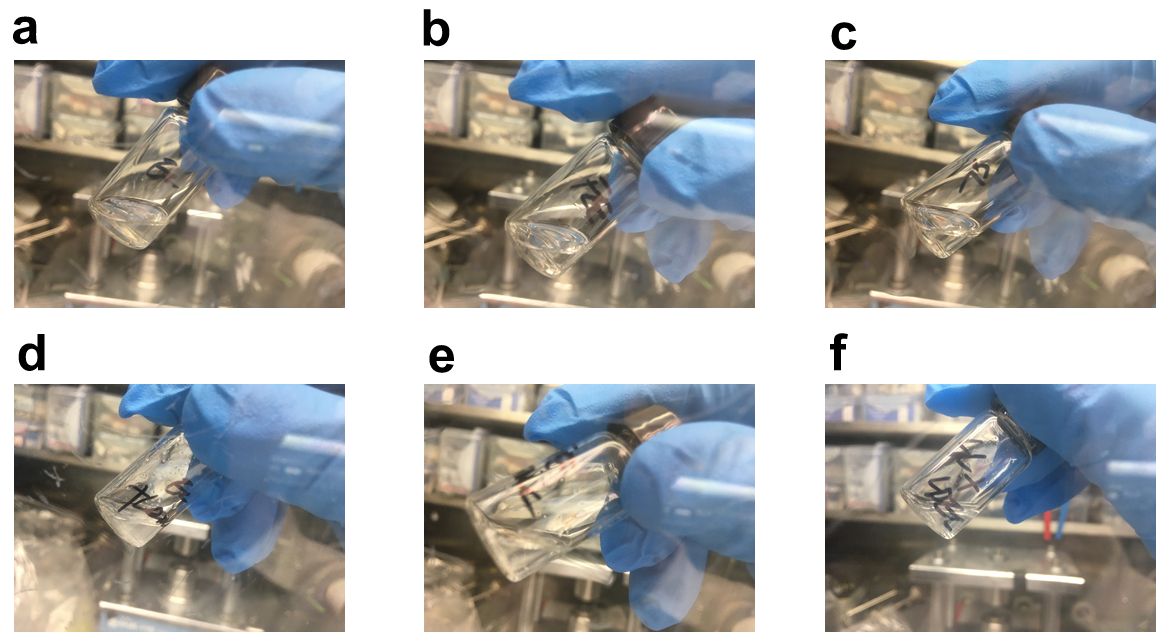
**

**Supplementary Figure 9**. The freshly prepared EE (**a**), FFT (**b**), and FST (**c**) electrolytes and after mixed with ~10 mg LiEMC, EE (**d**), FFT (**e**), and FST (**f**). The electrolyte amount used to dissolve the 10 mg LiEMC is ~ 0.5 mL. The large particles shown in the glass wall of (**d**) and (**e**) indicate the insoluble LiEMC in the corresponding electrolytes, while a rather clear solution in (**f**) corresponds to the partial dissolving of the organic LiEMC salt in the FST electrolytes.

**
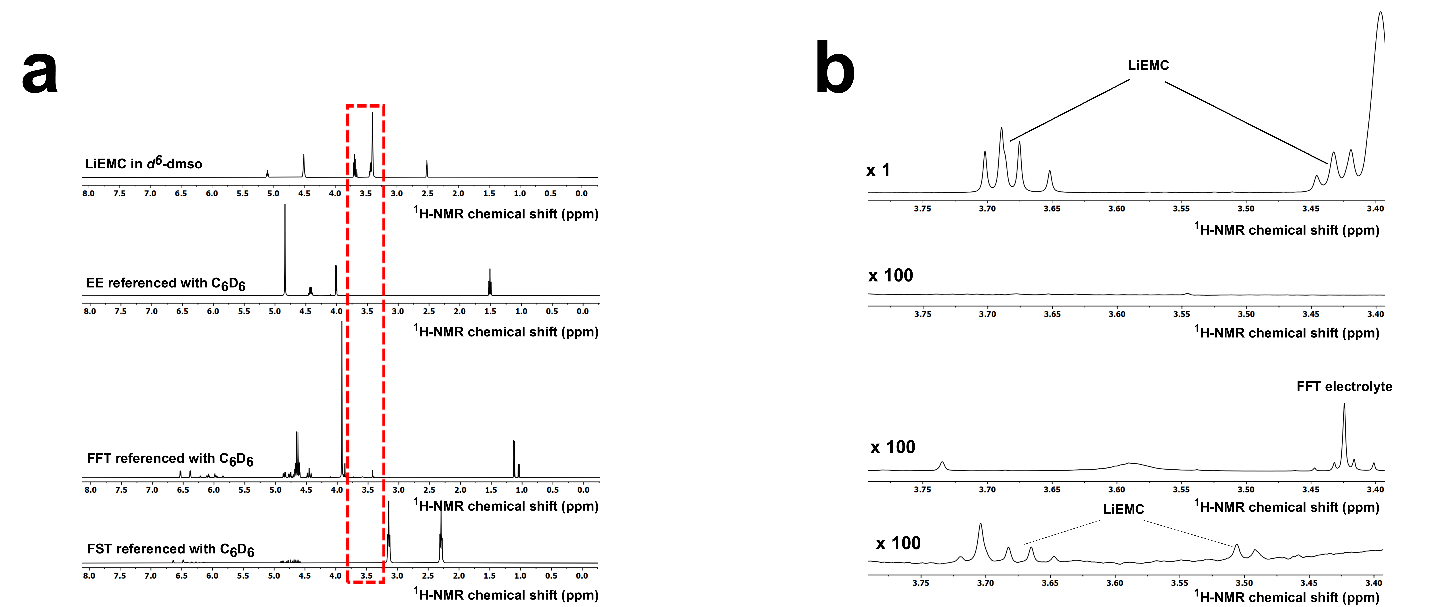
**

**Supplementary Figure 10**. ^1^H-NMR spectra for the solubility test of LiEMC in the studied electrolytes. (**a**) Full ^1^H-NMR spectra of standard LiEMC in *d^6^*-DMSO and EE, FFT, FST electrolytes after mixing with LiEMC with C_6_D_6_ as reference. (**b**) In the zoom-out view between 3.4 ppm to 3.8 ppm of the left spectra, a clear LiEMC signal was identified in the FST electrolytes while no obvious signal was observed in EE and FFT electrolytes.


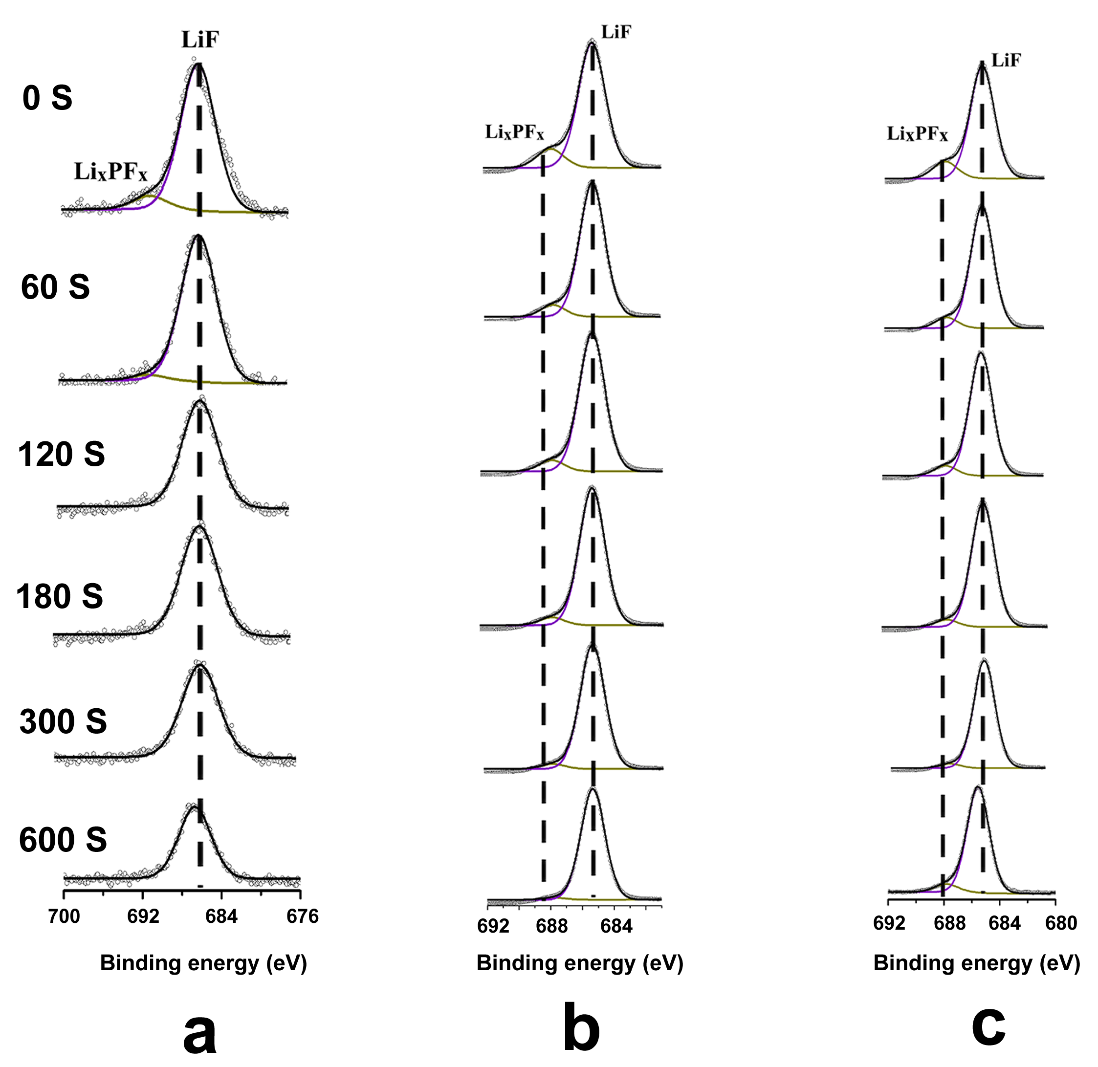


**Supplementary Figure 11**. XPS analysis of the SEI compositions on μSi anodes cycled with FST (**a**), FFT (**b**), and EE (**c**) electrolytes: The *F1s* spectra are displayed in columns, which show the corresponding depth profiling results at different sputtering times (0 s, 60 s, 120 s, 180 s, 300 s, 600 s). The *x*-axis represents the binding energy with the unit of eV, and the *y*-axis which shows the relative intensity with arbitrary units (a.u.) for all spectra was omitted for clarity.


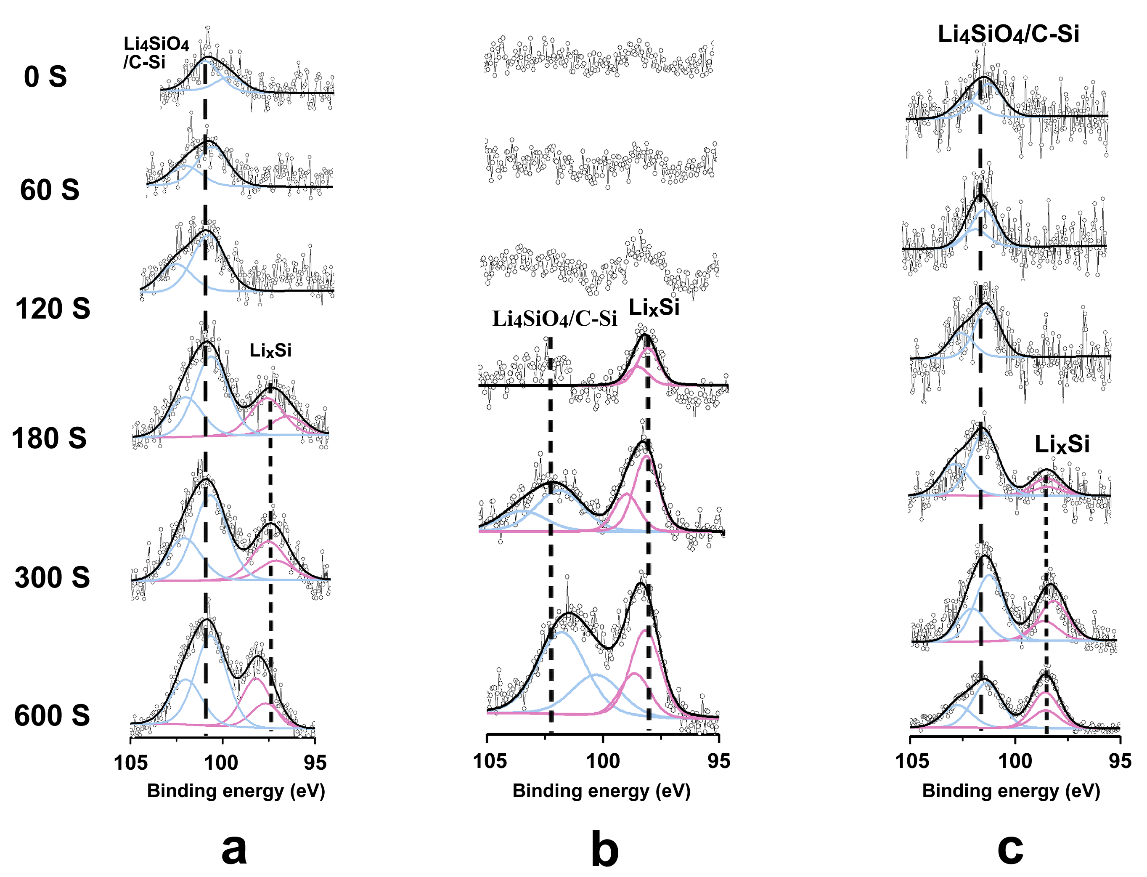


**Supplementary Figure 12**. XPS analysis of the SEI compositions on μSi anodes cycled with FST (**a**), FFT (**b**), and EE (**c**) electrolytes: The *Si2p* spectra are displayed in columns, which show the corresponding depth profiling results at different sputtering times (0 s, 60 s, 120 s, 180 s, 300 s, 600 s). The *x*-axis represents the binding energy with the unit of eV, and the *y*-axis which shows the relative intensity with arbitrary units (a.u.) for all spectra was omitted for clarity.


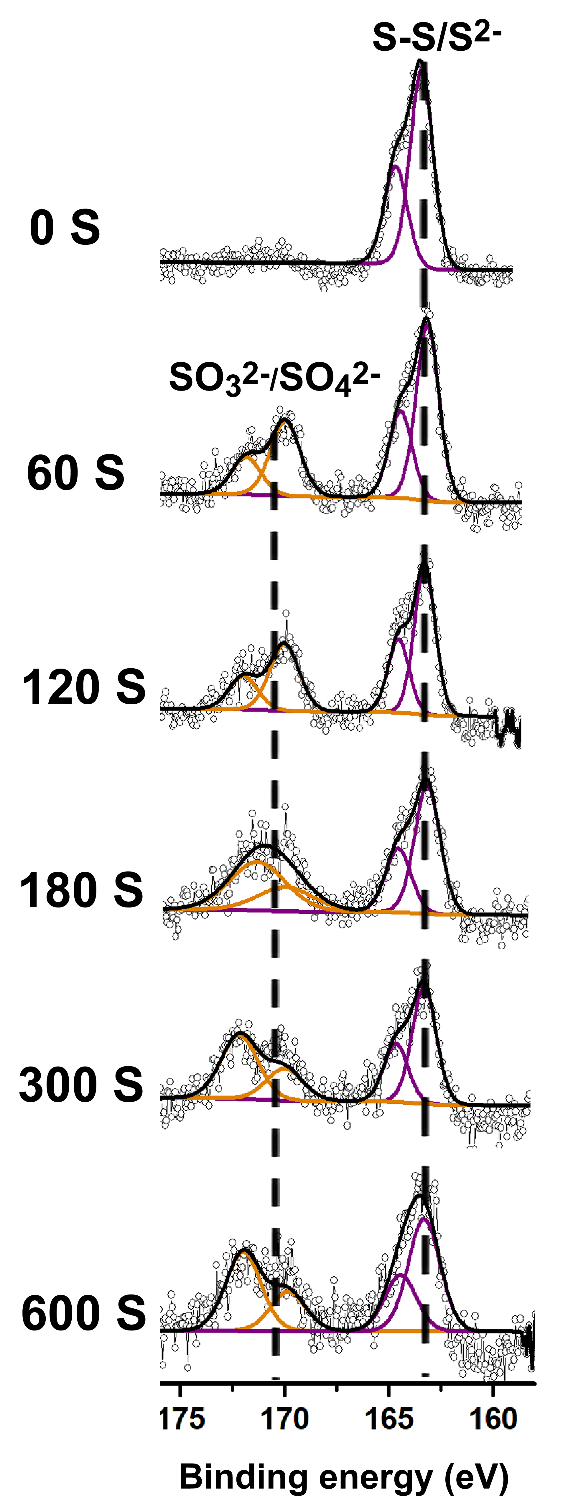


**Supplementary Figure 13**. XPS analysis of the SEI compositions on μSi anodes cycled with the FST electrolytes: The *S2p* spectra are displayed in columns, which show the corresponding depth profiling results at different sputtering times (0 s, 60 s, 120 s, 180 s, 300 s, 600 s). The *x*-axis represents the binding energy with the unit of eV, and the *y*-axis which shows the relative intensity with arbitrary units (a.u.) for all spectra was omitted for clarity.


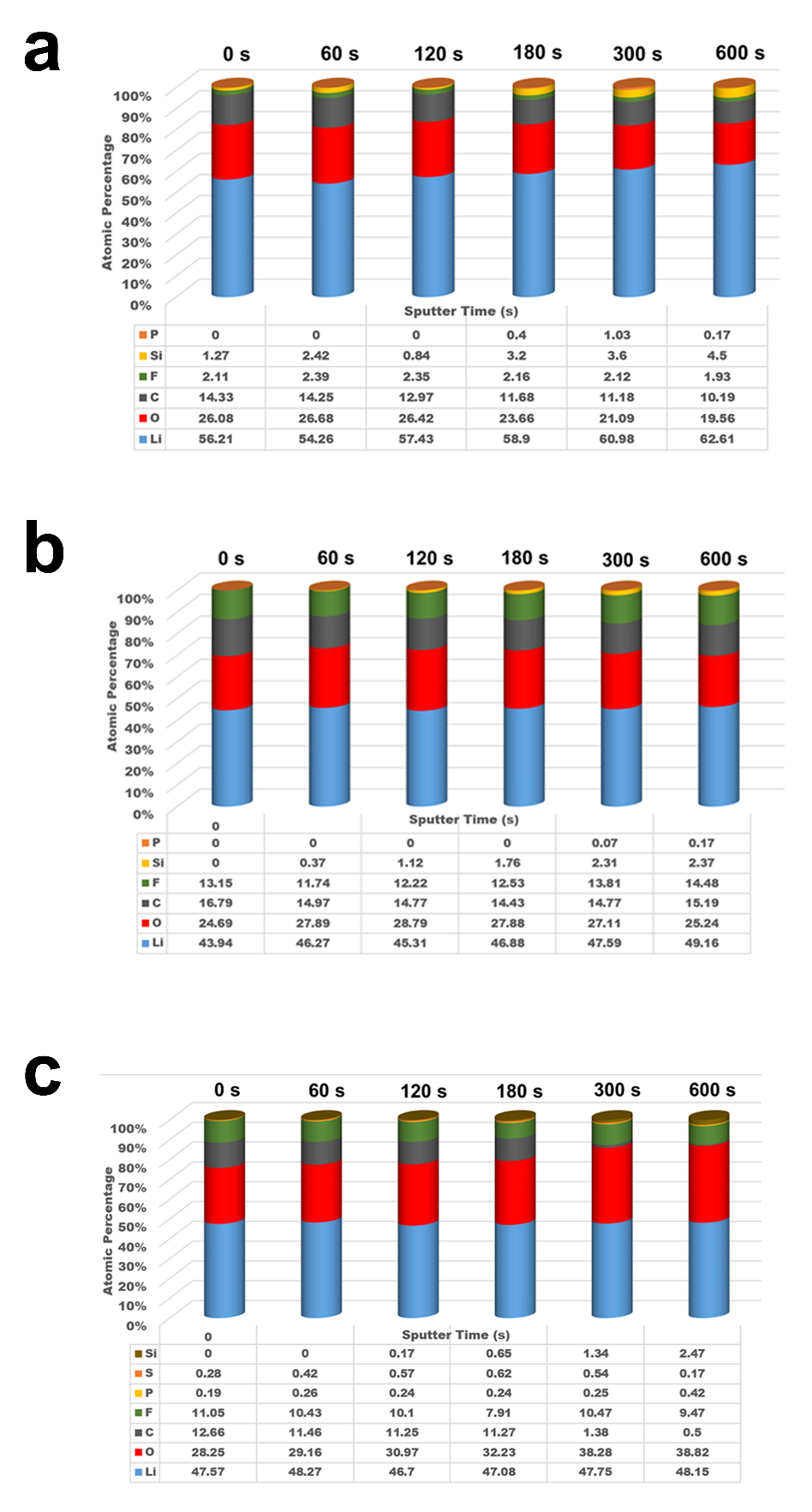


**Supplementary Figure 14**. Atomic compositions (Blue: Li, Red: O, Black: C, green: F, yellow: P, pink: S, dark grey: Si, after 0, 60, 120, 180, 300, 600s of Ar^+^ sputtering) of μSi electrodes cycled in different electrolytes, EE (**a**), FFT (**b**) and FST (**c**).

**
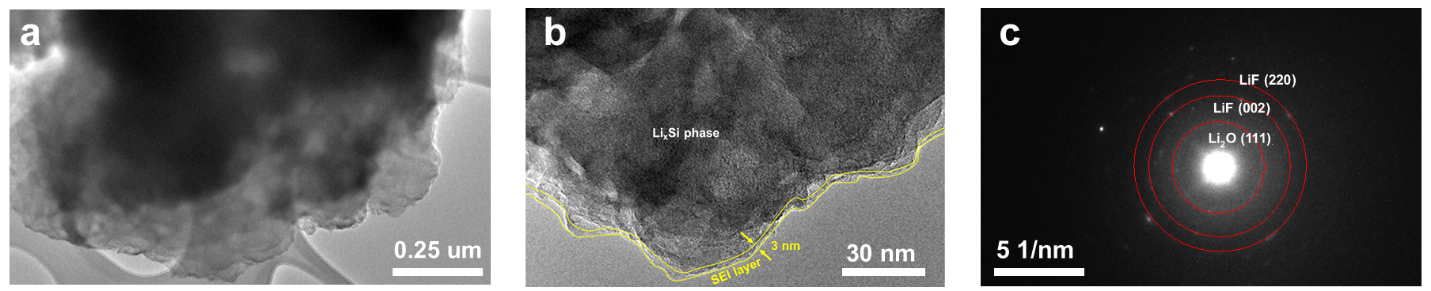
**

**Supplementary Figure 15**. High-resolution TEM images with different magnifications (**a** and **b**) and corresponding fast Fourier transform (**c**) of μSi electrode cycled in the FST electrolytes.

**
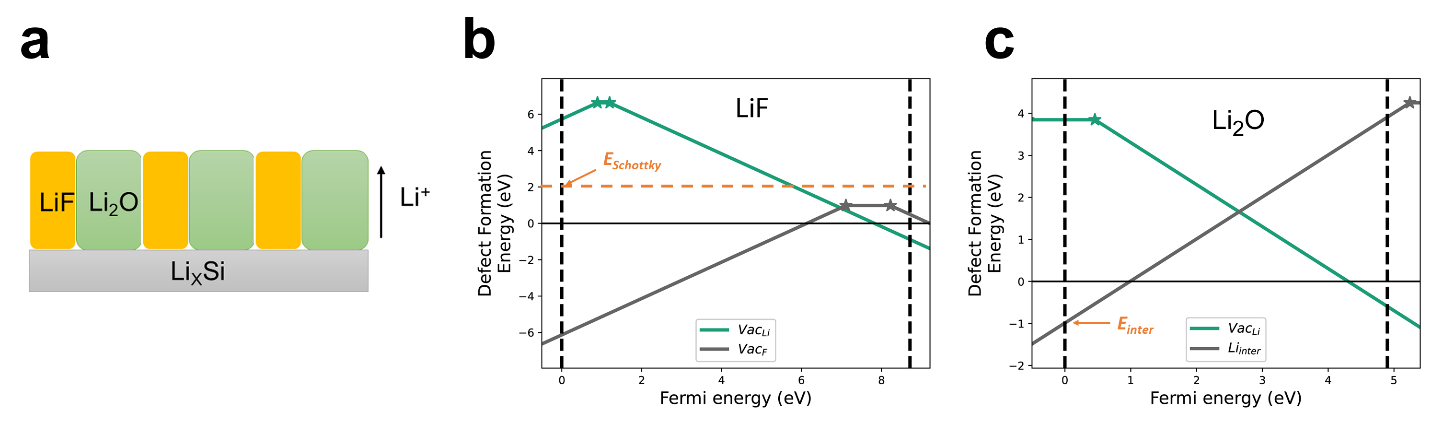
**

**Supplementary Figure 16**. Modeling of Li_2_O/LiF composite SEI on improving lithium-ion transport. (**a**) Cartoon demonstration of the simplified model where the topological distribution of the LiF and Li_2_O phases was set to be alternatively parallel so that the Li^+^ conduction path could penetrate along the SEI. (**b**) Formation energy vs Fermi level (referenced to the VBM) for the most favorable native defects in LiF and Li_2_O under lithium-rich (**µ_Li_** = 0) chemical potential conditions. Transitions are marked with stars. (**c**) The formation energy of Schottky defects was $E^{f}[{Schottky}_{LiF}^{0}]$=$E^{f}[{vac}_{Li}]$ + $E^{f}[{vac}_{F}]$ and its evolution with Fermi energy was plotted by a dashed orange line. Based on defect formation energy calculation, the dominant point defects of LiF and Li_2_O in equilibrium with the Li anode are Schottky defects and Li^+^ interstitial defects, respectively.


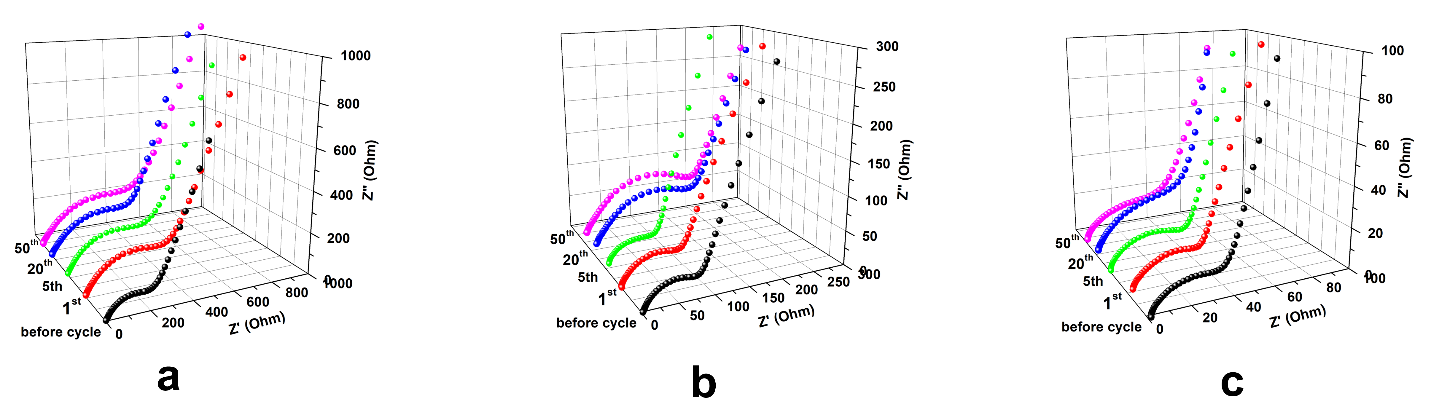


**Supplementary Figure 17**. Electrochemical impedance spectra of Li||μSi half cells with EE (**a**), FFT (**b**), and FST (**c**) electrolytes after different charge/discharge cycles.


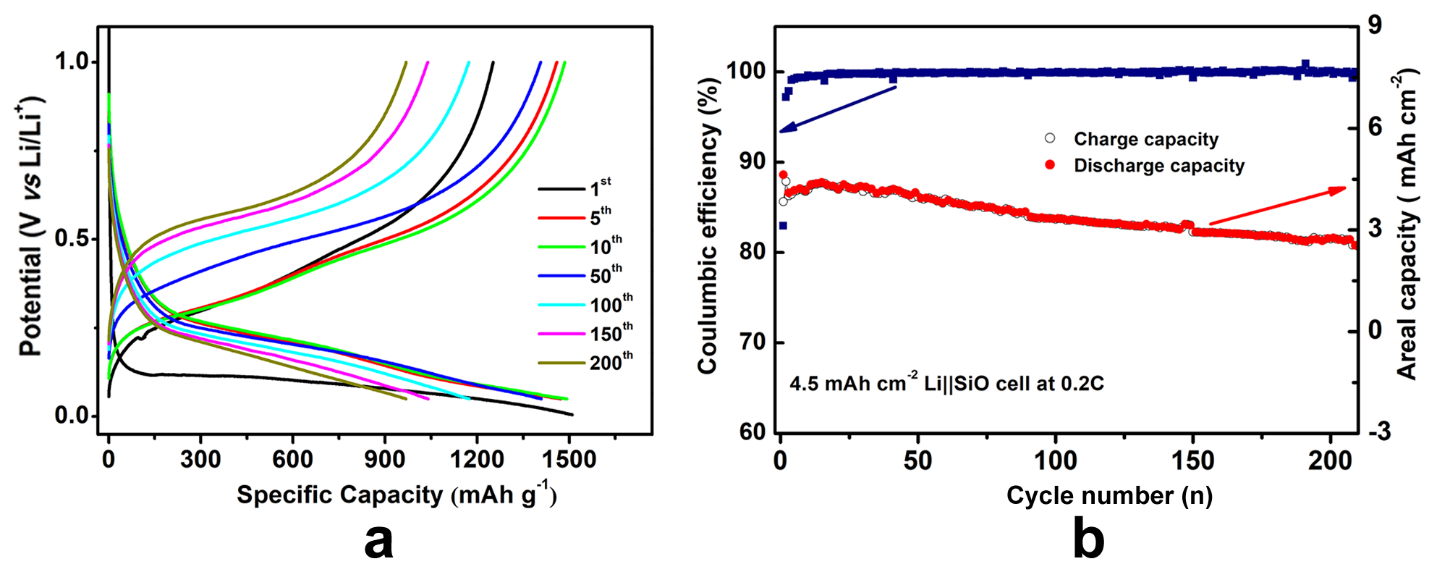


**Supplementary Figure 18**. Cycle performance of Li||SiO half cells using FST electrolytes. (**a**) Typical charge/discharge profiles and (**b**) Cycling stability and CE. The cell is first pre-cycled at 0.1C for one cycle before the long cycle at 0.2C. Source data are provided as a Source Data file.


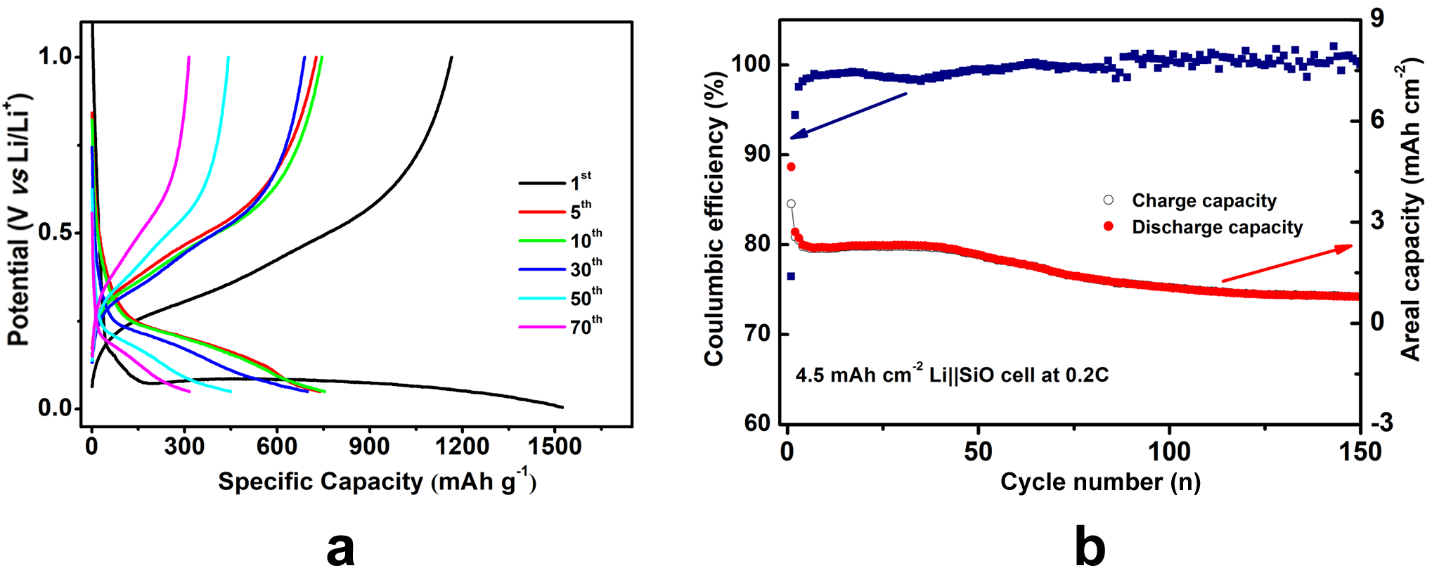


**Supplementary Figure 19**. Cycle performance of Li||SiO half cells using FFT electrolytes. (**a**) Typical charge/discharge profiles and (**b**) Cycling stability and CE. The cell is first pre-cycled at 0.1C for one cycle before the long cycle at 0.2C. Source data are provided as a Source Data file.


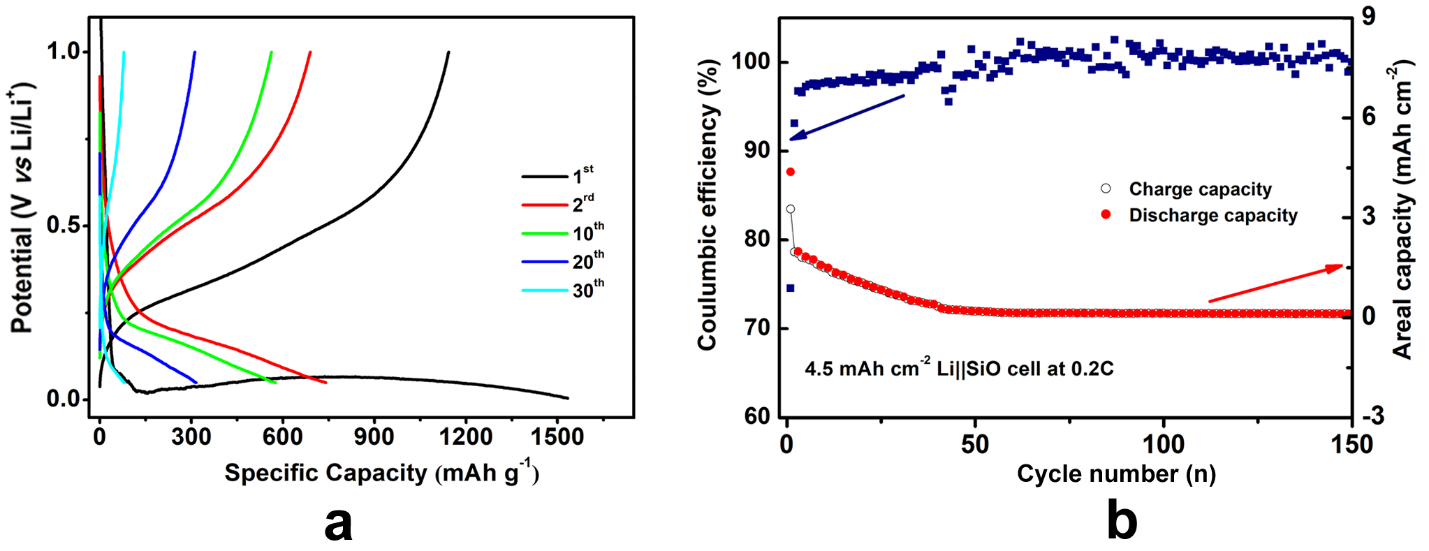


**Supplementary Figure 20**. Cycle performance of Li||SiO half cells using EE electrolytes. (**a**) Typical charge/discharge profiles and (**b**) Cycling stability and CE. The cell is first pre-cycled at 0.1C for one cycle before the long cycle at 0.2C. Source data are provided as a Source Data file.

**
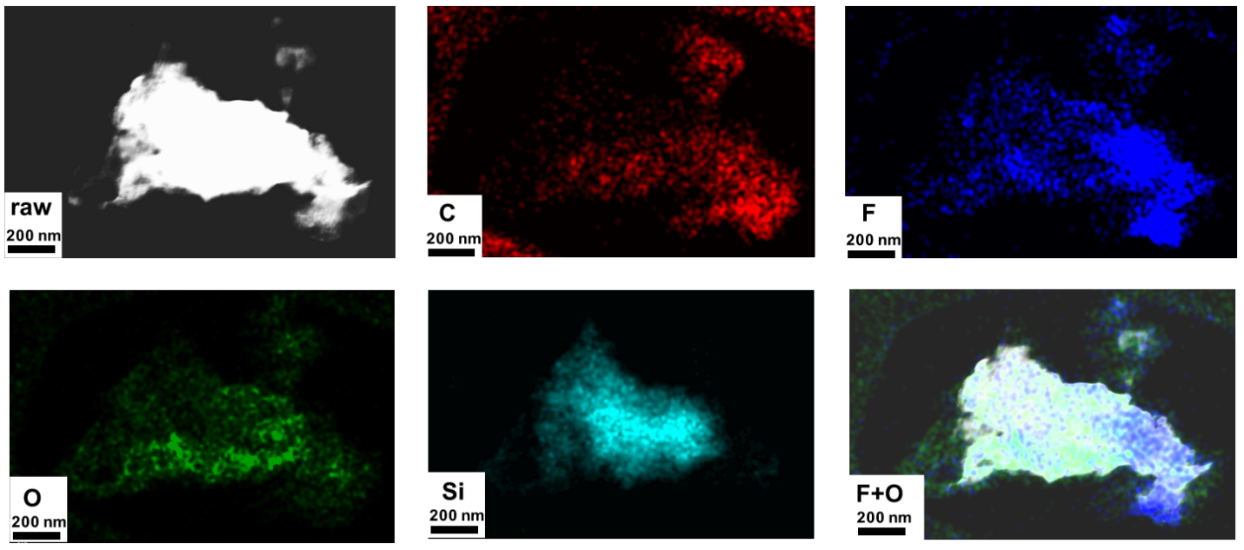
**

**Supplementary Figure 21**. Energy dispersive X-ray (EDX) mapping analysis of the μSi electrode composites after cycling with the FFT electrolytes, with C, F, O, and Si signals showing red, blue, green, and cyan color, respectively. The last photo shows the combination of F and O mapping, representing the LiF and Li_2_O distribution on the SiMPs surface.


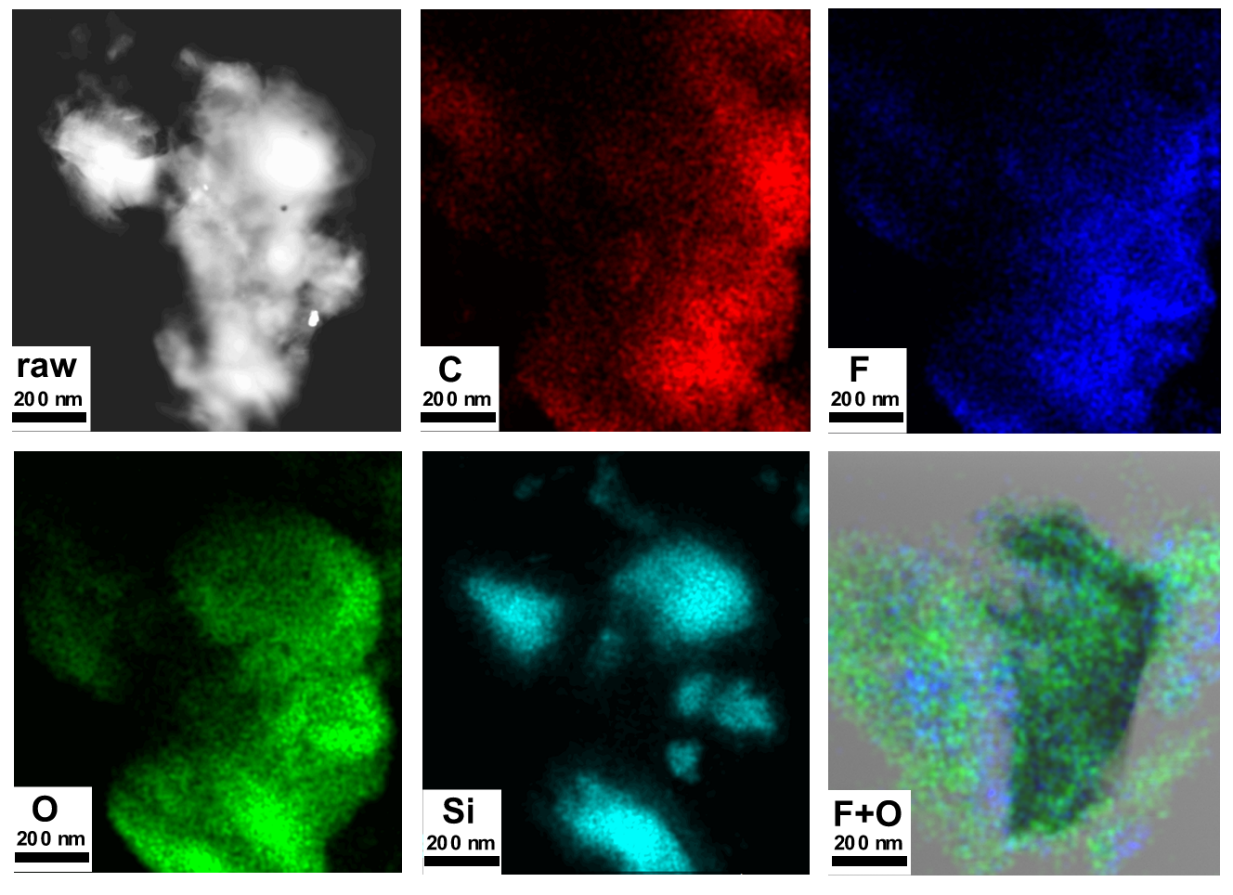


**Supplementary Figure 22**. Energy dispersive X-ray (EDX) mapping analysis of the μSi electrode composites after cycling with the FST electrolytes, with C, F, O, and Si signals showing red, blue, green, and cyan color, respectively. The last photo shows the combination of F and O mapping, representing the LiF and Li_2_O distribution on the SiMPs surface.


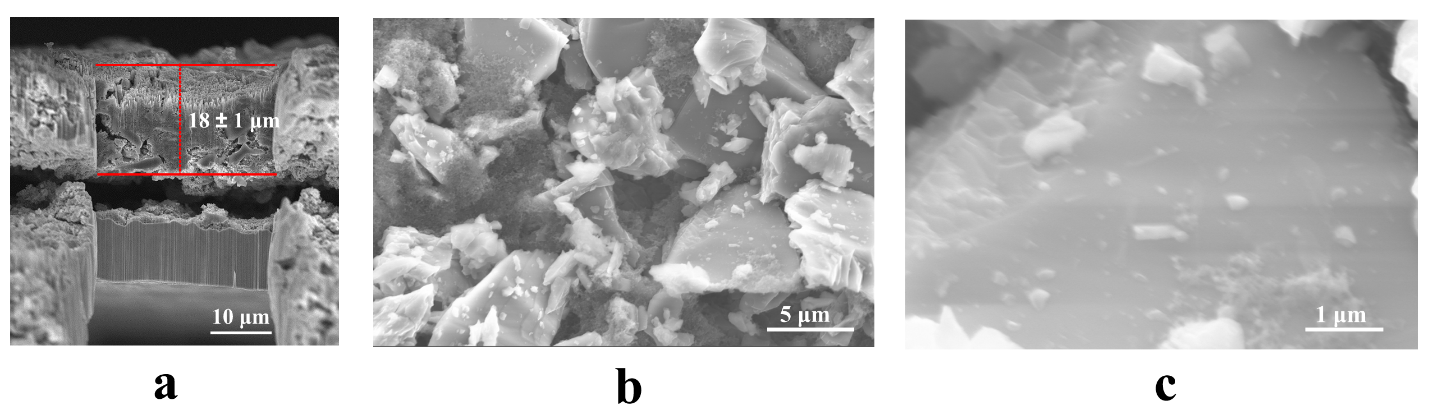


**Supplementary Figure 23**. The SEM images of the pristine 1-5 μSi electrodes: (**a**) the electrode thickness of pristine 4.1 mAh cm^-2^ μSi electrode, the error bar is defined as the average reading error from the electrode thickness measurement; (**b**) and (**c**) the micro-sized silicon particles in the freshly casted electrode.


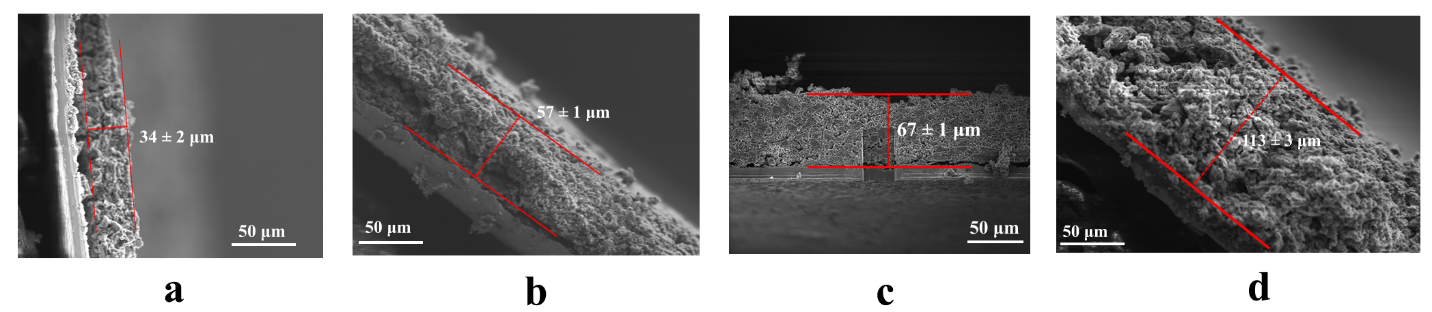


**Supplementary Figure 24**. The electrode thickness evolution under different cycle numbers with the EE electrolytes, the cell is stopped at the charged state to make these SEM images: (**a**) 1^st^ discharge, (**b**) 20^th^ discharge, (**c**) 50^th^ discharge, (**d**) 200^th^ discharge. The error bar is defined as the average reading error from the electrode thickness measurement.


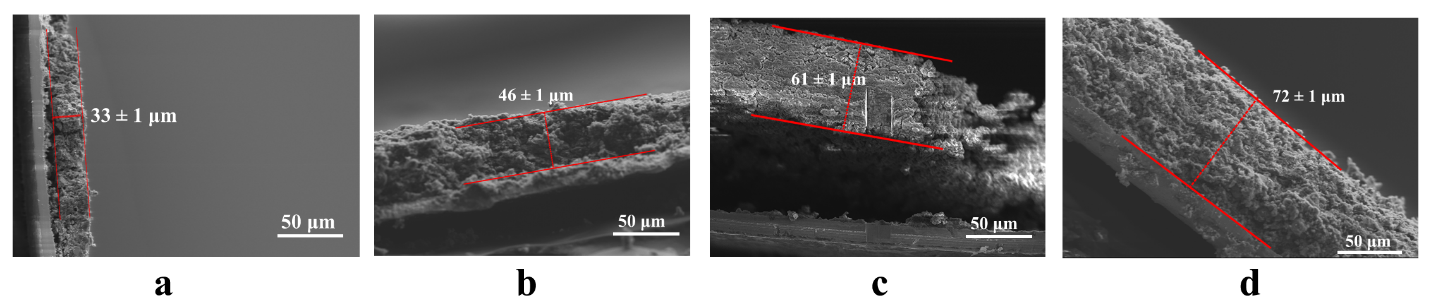


**Supplementary Figure 25**. The electrode thickness evolution under different cycle numbers with the FFT electrolytes, the cell is stopped at the charged state to make these SEM images: (**a**) 1^st^ discharge, (**b**) 20^th^ discharge, (**c**) 50^th^ discharge, (**d**) 200^th^ discharge. The error bar is defined as the average reading error from the electrode thickness measurement.


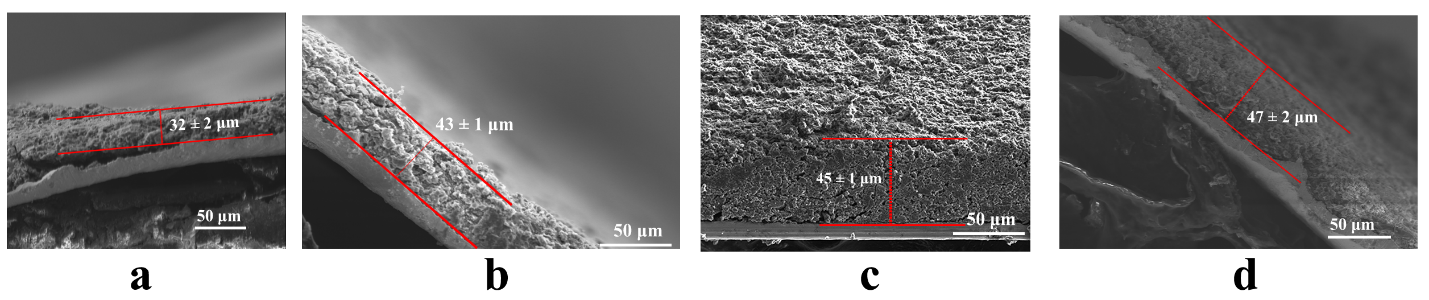


**Supplementary Figure 26**. The electrode thickness evolution under different cycle numbers with the FST electrolytes, the cell is stopped at the charged state to make these SEM images: (**a**) 1^st^ discharge, (**b**) 20^th^ discharge, (**c**) 50^th^ discharge, (**d**) 200^th^ discharge. The error bar is defined as the average reading error from the electrode thickness measurement.


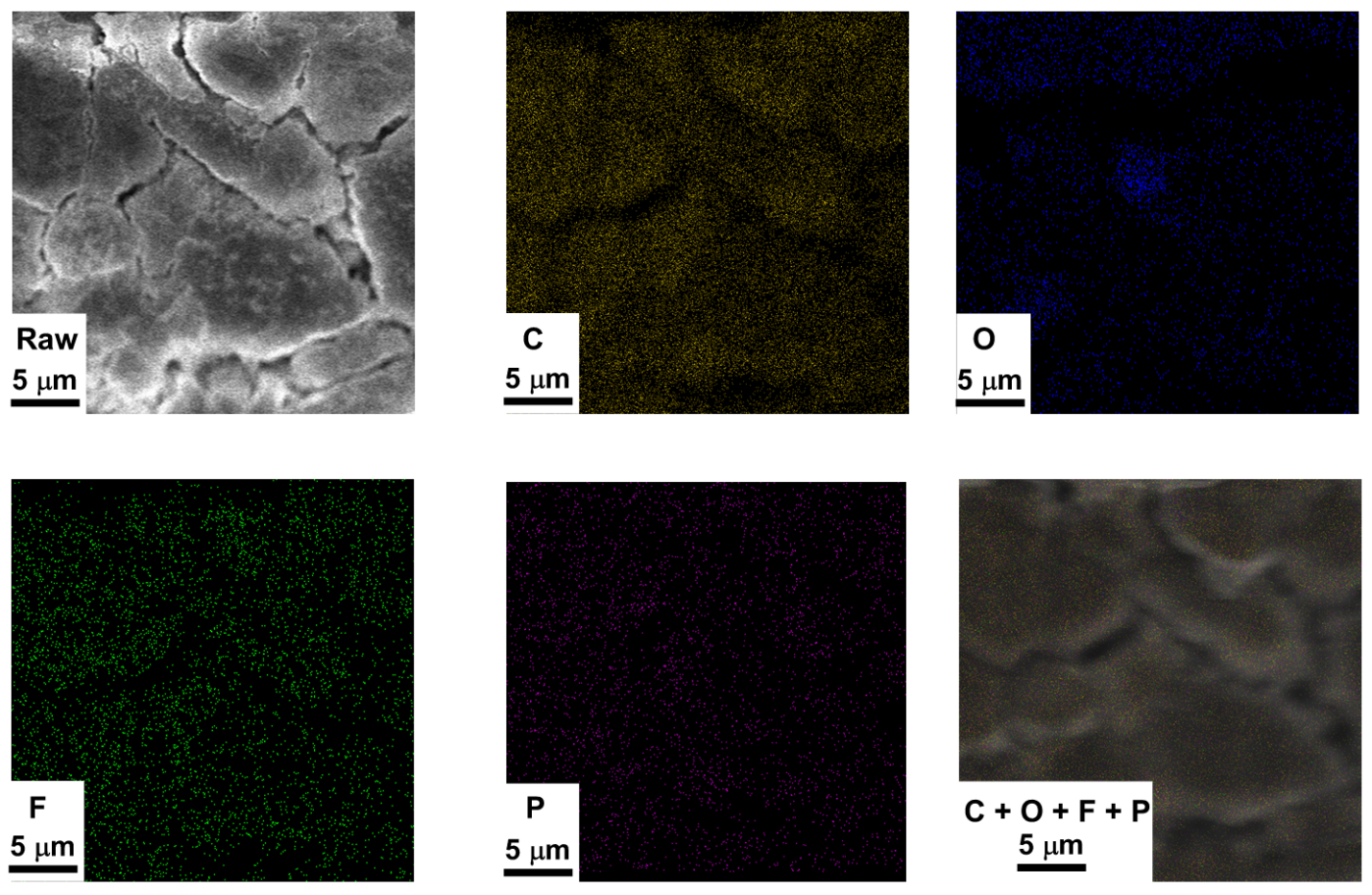


**Supplementary Figure 27**. **Morphology and energy dispersive X-ray (EDX) mapping analysis of Si particles after 200 cycles in FST electrolytes.** The C, O, F, and P signals are shown in yellow, blue, green, and magenta, respectively. The last photo shows the combination of C, O, F, and P mapping, representing the overview of SEI distribution on the SiMPs.


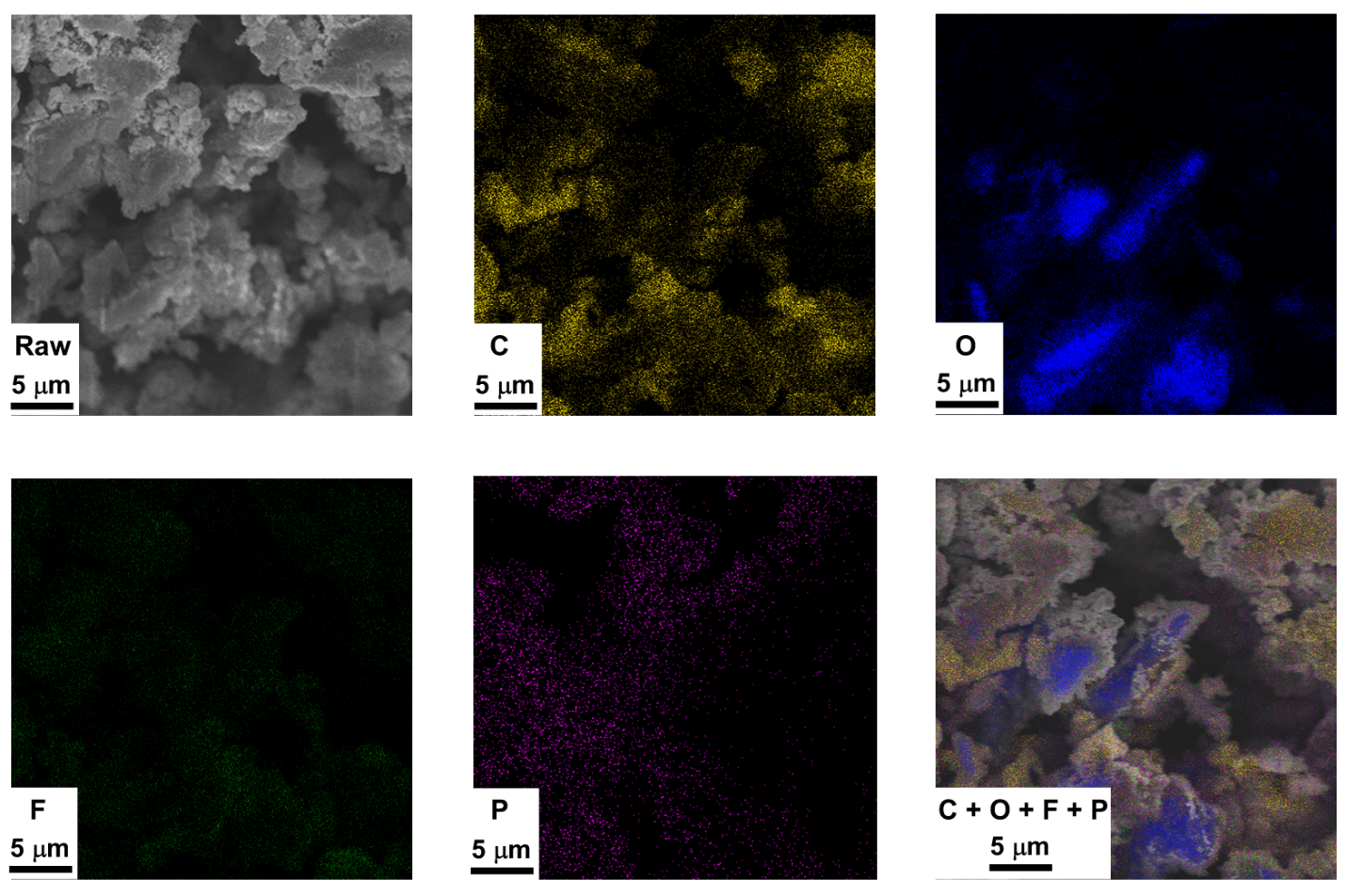


**Supplementary Figure 28**. **Morphology and energy dispersive X-ray (EDX) mapping analysis of Si particles after 200 cycles in FFT electrolytes.** The C, O, F, and P signals are shown in yellow, blue, green, and magenta, respectively. The last photo shows the combination of C, O, F, and P mapping, representing the overview of SEI distribution on the SiMPs.


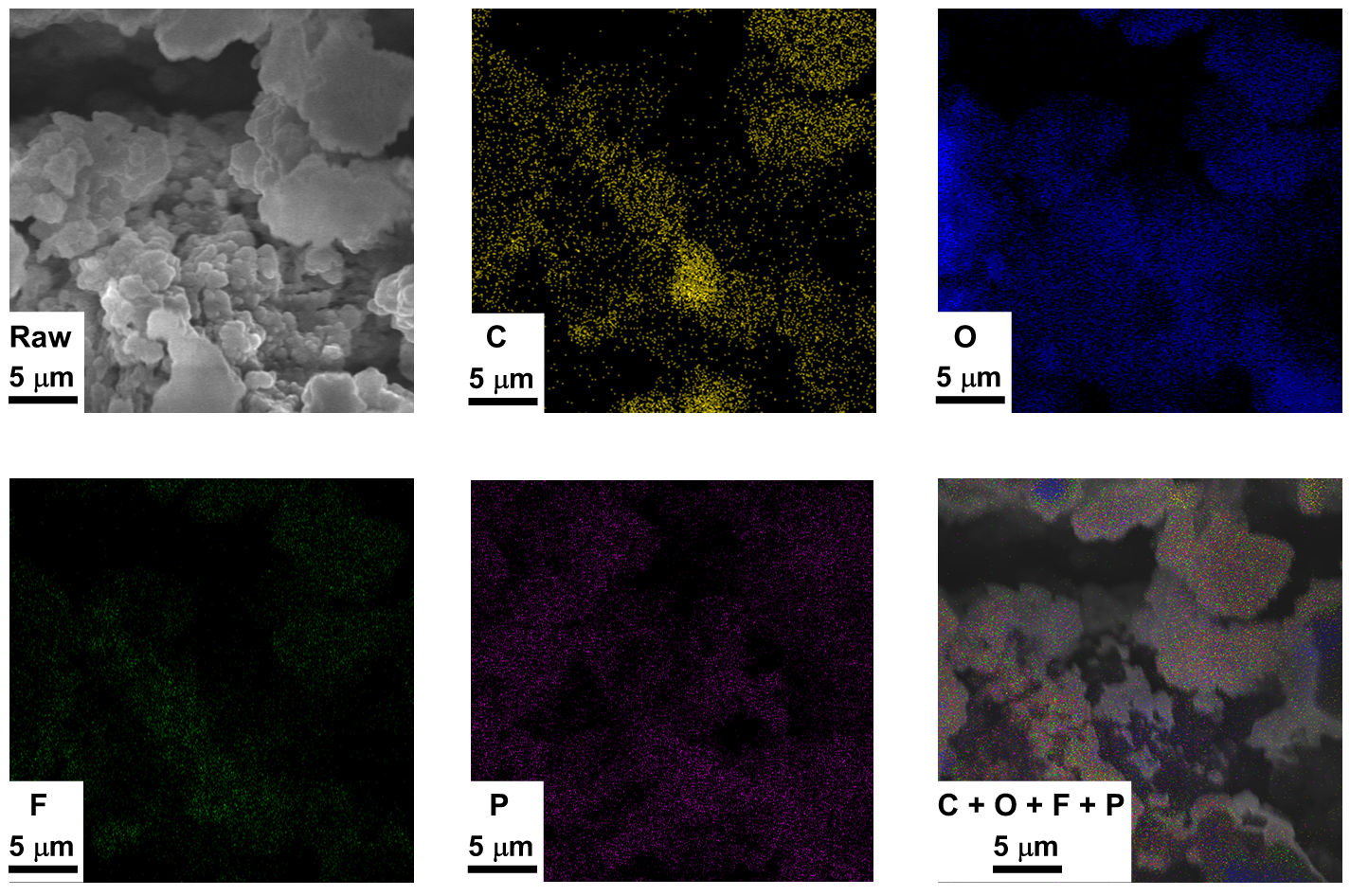


**Supplementary Figure 29**. **Morphology and energy dispersive X-ray (EDX) mapping analysis of Si particles after 200 cycles in EE electrolytes.** The C, O, F, and P signals are shown in yellow, blue, green, and magenta, respectively. The last photo shows the combination of C, O, F, and P mapping, representing the overview of SEI distribution on the SiMPs.


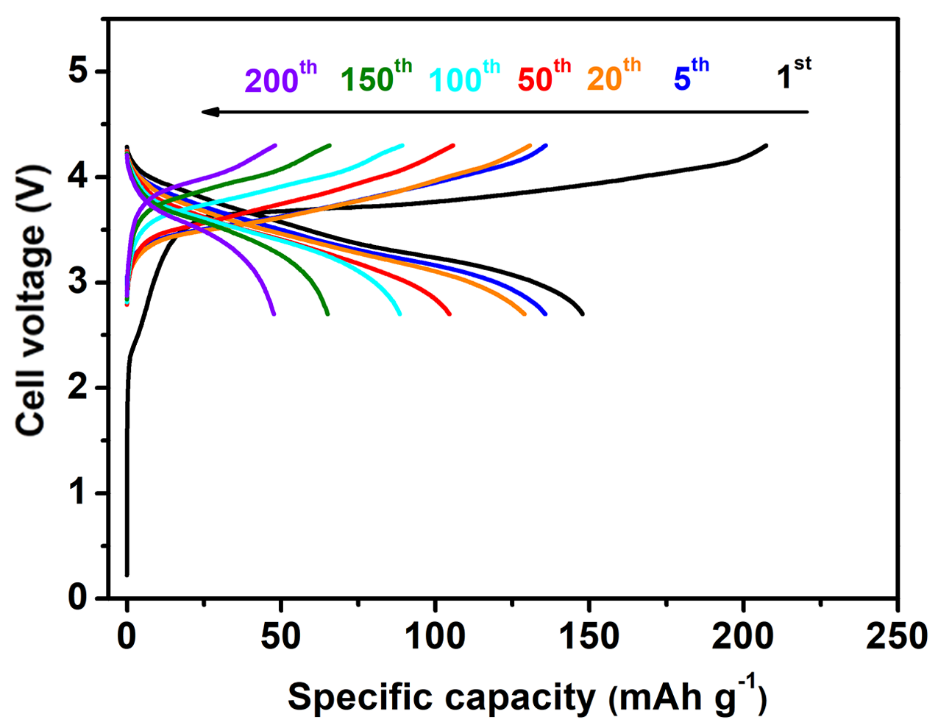


**Supplementary Figure 30**. Typical charge/discharge profiles of the µSi||NCA (4 mAh cm^-2^, N/P =1.1) coin cell in FFT electrolytes. The cycle rate is C/5 at room temperature with the first formation cycle at C/20. Source data are provided as a Source Data file.


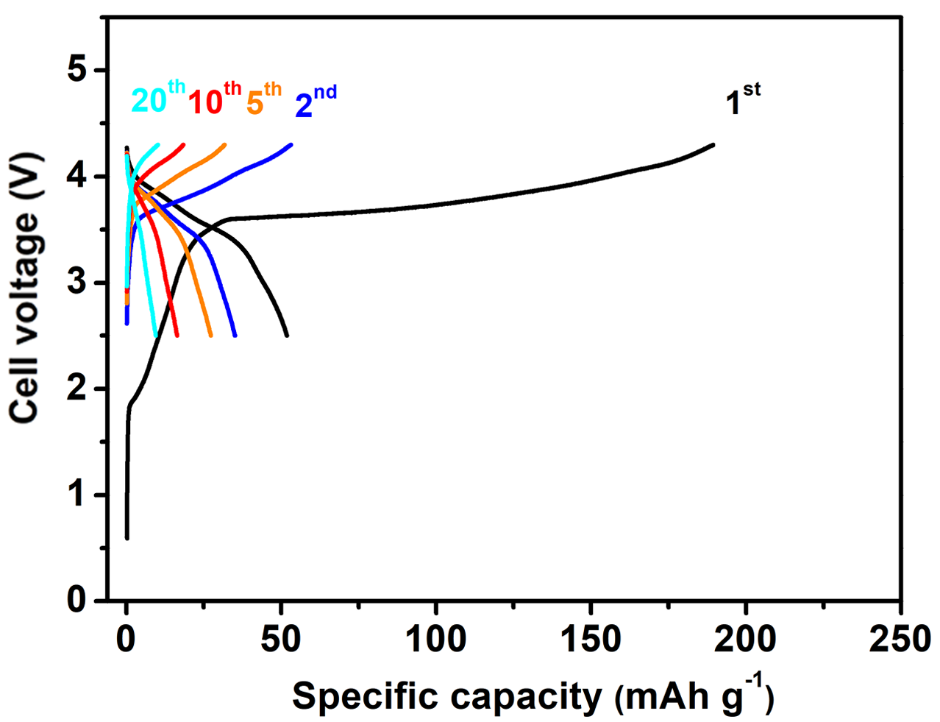


**Supplementary Figure 31**. Typical charge/discharge profiles of the µSi||NCA (4 mAh cm^-2^, N/P =1.1) coin cell in EE electrolytes. The cycle rate is C/5 at room temperature with the first formation cycle at C/20. Source data are provided as a Source Data file.

**
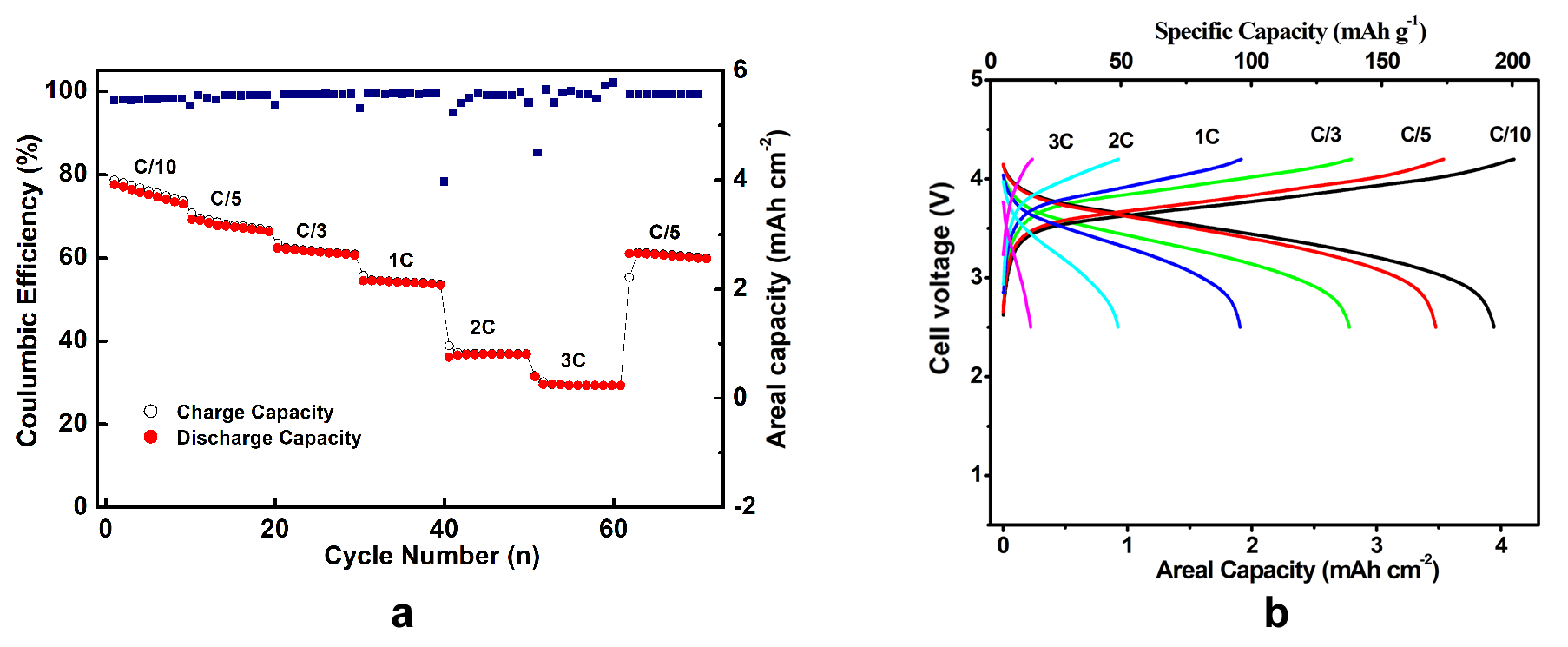
**

**Supplementary Figure 32**. Rate performance of µSi||NCA full cells in the FST electrolytes: (**a**) cycle rate, (**b**) rate voltage profiles. Source data are provided as a Source Data file.


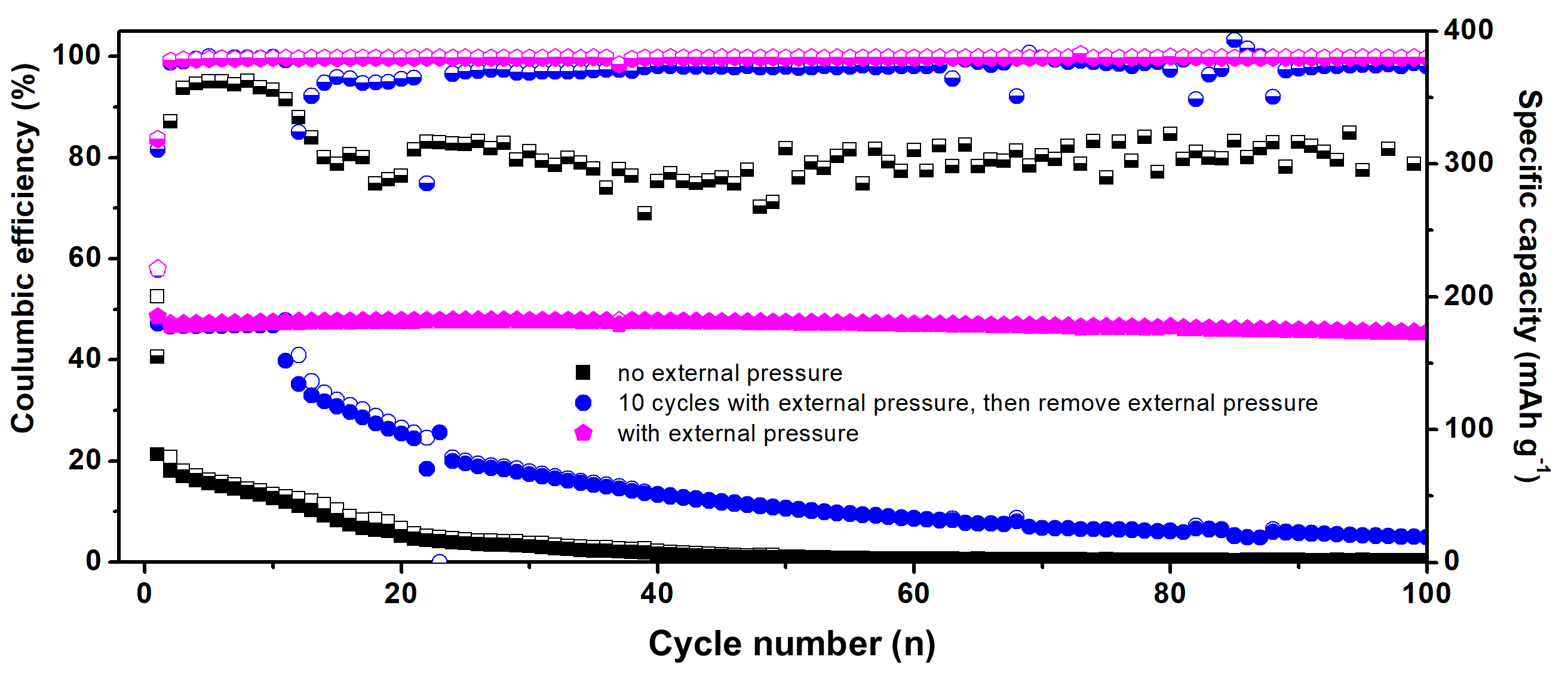


**Supplementary Figure 33**. The effect of external pressure on µSi||NCA (4 mAh cm^-2^, N/P = 1.1) pouch cell cycling. The μSi pouch cells were assembled with practical electrode loadings (~4 mAh cm^-2^ for NCA and ~4.1 mAh cm^-2^ for μSi, electrode size of ~2 cm by 2 cm) and cycled with FST electrolytes. Before cycling at C/5, one formation cycle at C/20 was conducted. The external pressure was applied with a steel presser at 0.1 MPa. Source data are provided as a Source Data file.

**
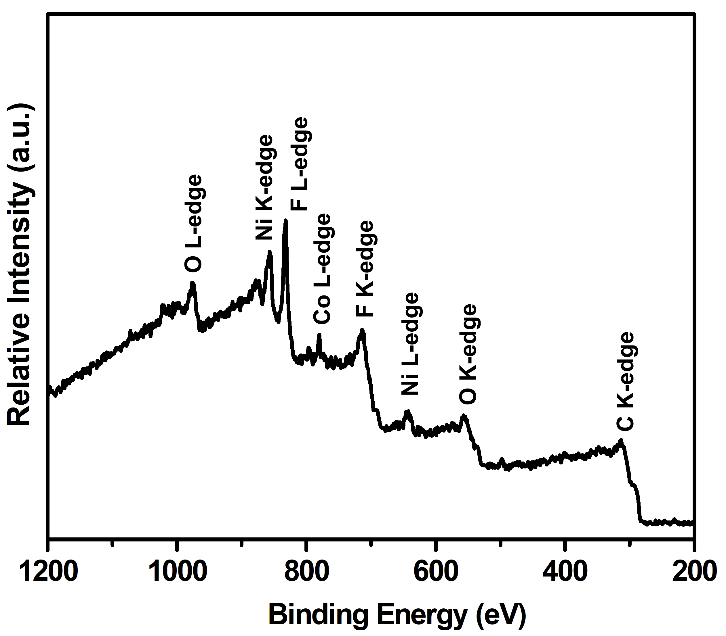
**

**Supplementary Figure 34**. XPS surface spectra of NCA cathode cycled in the FFT electrolytes in μSi||NCA full cells after 50 charge/discharge cycles.

**
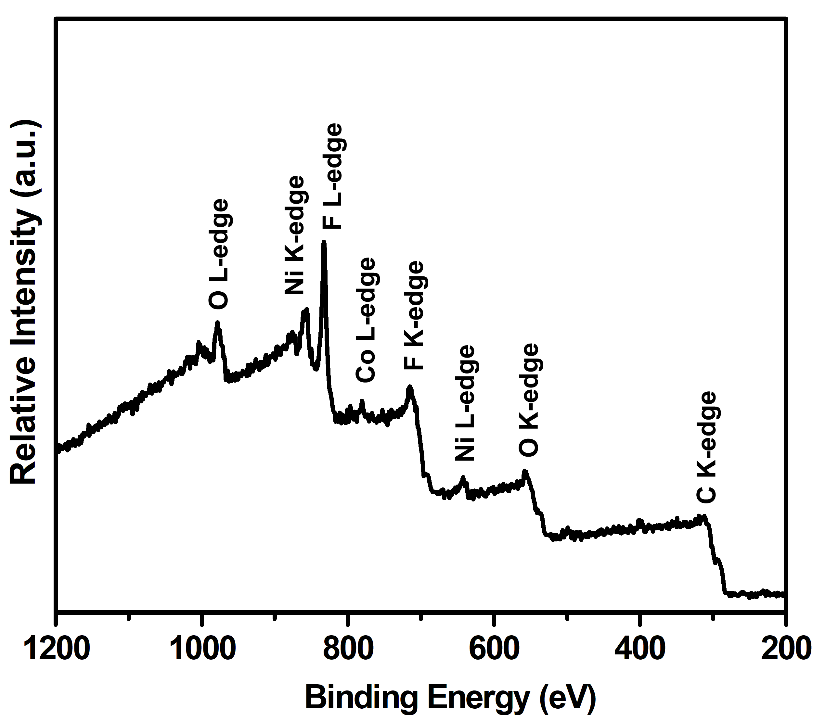
**

**Supplementary Figure 35**. XPS surface spectra of NCA cathode cycled in the FST electrolytes in μSi||NCA full cells after 50 charge/discharge cycles.

***4. Supplementary References***

1 Wu, M. *et al.* In situ formed Si nanoparticle network with micron-sized Si particles for lithium-ion battery anodes. *Nano Lett.* **13**, 5397–5402 (2013).

2 Li, Y. *et al.* Growth of conformal graphene cages on micrometer-sized silicon particles as stable battery anodes. *Nat. Energy* **1***,* 1–9 (2016).

3 Choi, S. *et al.* Highly elastic binders integrating polyrotaxanes for silicon microparticle anodes in lithium-ion batteries. *Science* **357**, 279–283 (2017).

4 Xu, Z. *et al.* Silicon Microparticle Anodes with Self-Healing Multiple Network Binder. *Joule* **2**, 950–961 (2018).

5 Wang, C. *et al.* Self-healing chemistry enables the stable operation of silicon microparticle anodes for high-energy lithium-ion batteries. *Nat. Chem.* **5**, 1042–1048 (2013).

6 Chen, J. *et al.* Electrolyte design for LiF-rich solid–electrolyte interfaces to enable high-performance microsized alloy anodes for batteries. *Nat. Energy* **5**, 386–397 (2020).

7 Li, Z. *et al.* Nonvolatile and Nonflammable Sulfolane-Based Electrolyte Achieving Effective and Safe Operation of the Li–O2 Battery in Open O2 Environment. *Nano Lett.* **22**, 815–821 (2022).

8 Le, P. M. L. *et al.* Electrochemical performance of sulfone-based electrolytes in sodium ion battery with NaNi1/3Mn1/3Co1/3O2 layered cathode. *Sci. Technol. Developm. J.* **22**, 335–342 (2019).

9 Xu, K. *et al.* Nonflammable Electrolytes for Li-Ion Batteries Based on a Fluorinated Phosphate. *J. Electrochem. Soc.* **149**, A1079 (2002).

10 Nagasubramanian, G. *et al.* Hydrofluoroether electrolytes for lithium-ion batteries: Reduced gas decomposition and nonflammable. *J. Power Sources* **196**, 8604–8609 (2011).

11 Wang, L. *et al.* Identifying the components of the solid-electrolyte interphase in Li-ion batteries. *Nat. Chem.* **11**, 789–796 (2019).

12 Zorko, M. *et al.* doi:10.21203/rs.3.rs-1950688/v1 (2022)

13 Jain, A. *et al.* A high-throughput infrastructure for density functional theory calculations. *Comput. Mater. Sci.* **50**, 2295–2310 (2011).

14 Broberg, D. *et al.* PyCDT: A Python toolkit for modeling point defects in semiconductors and insulators. *Comput. Phys. Commun.* **226**, 165–179 (2018).

15 Pan, J. *et al.* General method to predict voltage-dependent ionic conduction in a solid electrolyte coating on electrodes. *Phys. Rev. B* **91**, 134116 (2015).

16 Maier, J. Space charge regions in solid two-phase systems and their conduction contribution—I. Conductance enhancement in the system ionic conductor-‘inert’phase and application on AgCl: Al_2_O_3_ and AgCl: SiO_2_. *J. Phys. Chem. Solids*, **46**, 309–320 (1985).

17 Benitez, L. *et al.* Ion Diffusivity through the Solid Electrolyte Interphase in Lithium-Ion Batteries. *J. Electrochem. Soc.* **164**, E3159–E3170 (2017).
