# Supplementary material for: High voltage electrolytes for lithium-ion batteries with micro-sized silicon anodes
Source: Nat Commun. 2024 Feb 8;15:1206. doi: 10.1038/s41467-024-45374-0 (PMC10853533; doi:10.1038/s41467-024-45374-0)
Supplement: Supplementary file 3 — Description of Additional Supplementary Files [file 41467_2024_45374_MOESM3_ESM.pdf]

Description of Additional Supplementary Files

Supplementary Movie 1: Flammable test video for the EE electrolytes

Supplementary Movie 2: Flammable test video for the FFT electrolytes

Supplementary Movie 3: Flammable test video for the FST electrolytes
