# Supplementary material for: High voltage electrolytes for lithium-ion batteries with micro-sized silicon anodes
Source: Nat Commun. 2024 Feb 8;15:1206. doi: 10.1038/s41467-024-45374-0 (PMC10853533; doi:10.1038/s41467-024-45374-0)
Supplement: Supplementary file 7 — Source Data [file 41467_2024_45374_MOESM7_ESM.zip › source code file.zip!/code source file/FST electrolytes_MD/Description_of_MD_files.pdf]

Description of the force field file for the Molecular Dynamics (MD) simulation package.
The description was largely written by Justin Hooper (Wasatch Molecular Inc.) and Oleg Borodin.

ff.dat

The `ff.dat` file is comprised of seven sections that contain the necessary information to completely specify the force field determining inter-atomic interactions between the species present in the simulation. The `ff.dat` file controls both non-bonded and bonded interactions, including dispersion, charge-charge interactions, polarizability, bonds, bends, torsions, and improper torsions.

Section 1
Number of non-bonded types

The first data line in the `ff.dat` file looks similar to the following:

```
* Number of repulsion-dispersion types (T_RD)
  9
```

The number of repulsion-dispersion types (T_{RD}) is the number of atomic species with unique repulsion-dispersion interactions within the system. This number should be less than or equal to the parameter *maxtypes* in the `params.h` file.

Section 2
Non-bonded parameters

Non-bonded interactions are represented by the combination of a repulsion-dispersion interaction, a coulomb interaction term representing the two-body electrostatic potential, and a many-body polarizability term:

$$U^{NB}(r_{ij}) = U^{RD}(r_{ij}) + U^{Coul}(r_{ij}) + U^{Pol}(\vec{r})$$

$$U^{RD}(r_{ij}) = A\exp(-Br_{ij}) - Cr_{ij}^{-6} + W\left(\frac{12}{Br_{ij}}\right)^{12}$$

$$U^{Coul}(r_{ij}) = \frac{1}{2}\sum_i\sum_j \frac{q_i q_j}{4\pi\varepsilon_0 r_{ij}}$$

$$U^{Pol}(\vec{r}) = \sum_i \vec{\mu}_i \vec{E}_i^0 - \frac{1}{2}\sum_i\sum_j \vec{\mu}_i \overset{\leftrightarrow}{T} \vec{\mu}_j + \sum_i \frac{\vec{\mu}_i \vec{\mu}_j}{2\alpha_i}$$

The lines following the number of dispersion types declaration set out the interaction parameters for the dispersion interactions. At a minimum, there must be at least T_{RD} lines, each of which contain a full set of dispersion parameters for the indicated type. A full set of repulsion-dispersion parameters for a single repulsion-dispersion type contains the following terms:

```
*~~~~~~~~~~~~~~~~~~~~~~~~~~~~~~~~~~~~~~~~~~~~~~~~~~~~~~~~~~~~~~~~~~~~~~~~~~~~
* Label    A           B          C          D      Mass    Notes
*   Charge      Induced polarizability
*~~~~~~~~~~~~~~~~~~~~~~~~~~~~~~~~~~~~~~~~~~~~~~~~~~~~~~~~~~~~~~~~~~~~~~~~~~~~
Cm         108283.00  3.64050    560.53     0.00   12.011  exp-6  |8. Cm
```

Parameter	Units	Variable Type
Label	None	Character*3
A	kcal/mol	Double precision
B	Å^{-1}	Double precision
C	Å^{6}·kcal/mol	Double precision
Mass	g/mol	Double precision

The *label* is a 3 character tag used to denote the specific atom type for future use in the coordinates files.

The *A* and *B* parameters collectively define the repulsive barrier for the non-bonded interactions between two atoms of the labeled types, while the *C* parameter describes the attractive tail of the potential. The atomic *mass* is also specified here. The remainder of the line after the *mass* specification may be utilized for notes about the species described. The parameter labeled *D* is no longer utilized in the current forcefield, and should be set to 0.0.

In addition to the dispersion parameters, it is also possible to specify one or a number of charge permutations for the base atom. In order to specify a charge permutation associated with a set of dispersion parameters, the lines following the description of the dispersion parameters are appended with a line for each different charge species present. These charge description lines are required to start with at least one space, after which the static *charge* and *induced polarizability* of the species is listed:

```
*~~~~~~~~~~~~~~~~~~~~~~~~~~~~~~~~~~~~~~~~~~~~~~~~~~~~~~~~~~~~~~~~~~~~~~~~~~~~
~
* Label    A           B          C          D      Mass       Notes
*  Charge       Induced polarizability
*~~~~~~~~~~~~~~~~~~~~~~~~~~~~~~~~~~~~~~~~~~~~~~~~~~~~~~~~~~~~~~~~~~~~~~~~~~~~
~
Cm         108283.00  3.64050    560.53     0.00   12.011     exp-6 |8. Cm
   -0.0929      0.6500     |Nitrogen bonded methyl carbon in im+, pyr+
   -0.0764      1.0500     |Terminal methyl carbon in alkyl chain
```

Parameter	Units	Variable Type
Charge (q_i)	e	Double precision
Induced polarizability (α_i)	Å^{3}	Double precision

The *charge* represents the total static charge of the given species, while the *induced polarizability* represents the relative ability of local electric field gradients to further polarize the charge at the atom. The remainder of the line is available for notes regarding what the particular charge species represents.

The above snippet of the `ff.dat` file defines two atom types. Both have dispersion parameters that represent a methyl carbon. The first atom type has a static charge of -0.0929e, an induced polarizability of 0.6500 Å^3, and represents an ammonium bonded methyl group while the second has a static charge of -0.0764e, an induced polarizability of 1.0500 Å^3, and represents a carbon bonded methyl group. Both atom types share the label Cm, with the different charge types being represented in the `coords.inp` file by the addition of a two-integer charge index to differentiate the different charge species (see the description of `coords.inp` for further information).

In addition to combining rules, it is possible to specify non-default cross-terms to better describe the interaction between two different atom types. The non-default cross-terms immediately follows the dispersion parameter section and has the following format:

```
*                             non-default cross terms
*Label1_Label2        A          B          C          D
* ------------------------------------------------------------------
Cm _C                 102702.04  3.63127    539.82     0.00
Cm _N+                52447.87   3.93375    261.55     0.00
…
N  _O=                23978.15   3.45339    446.39     0.00
!
```

Parameter	Units	Variable Type
Label1_Label2	None	Character*7
A	kcal/mol	Double precision
B	Å^{-1}	Double precision
C	Å^6·kcal/mol	Double precision

Lucretius™ parses the appropriate cross-term interaction by using the value of *Lbl1_Lbl2* to form the appropriate index into the interaction matrix. As such, it is critical that both *Lbl1_Lbl2* be exactly 7 characters in length, with the first three characters comprising one full label (including any trailing spaces necessary to reach 3 characters total) from the preceding section, the fourth character is the underscore ("_"), and the fifth through seventh characters comprising a second, differing full label from the preceding section. The values of *A*, *B*, and *C* have the same meanings and units as in the preceding section, except here they dictate the interaction between two atoms of differing types. As in the homoatomic section, the D parameter is no longer used and should always be set as 0.0. The non-default cross term section is terminated by a line containing a single ! as the first character on the line.

Section 3

Bonding Potentials

The bond energy between any two contiguous atoms employs a harmonic oscillator model:

$$U_{Bond} = \frac{K_{ij}^{Bond}}{2}\left(r_{ij} - r_{ij}^{0}\right)^2$$

The first line of the bond parameterization subsection of the `ff.dat` file dictates the number of unique bond types (T_{Bond}) present in the system:

```
*                                              Bonds
   14
```

The remaining T_{Bond} lines in the bonded energy subsection describe the individual bonds in ascending order, indexed from 1 to T_{Bond}. Each line consists of a (informative only) label, as well as three parameters representing the bond interactions. Mapping of atoms to a specific bond type is accomplished in the `connectivity.dat` file.

```
*~~~~~~~~~~~~~~~~~~~~~~~~~~~~~~~~~~~~~~~~~~~~~~~~~~~~~~~~~~~~~~~~~~~~~~~~~~~~
* Label_ij         K_Bond     R0         R0 (constrained)
*~~~~~~~~~~~~~~~~~~~~~~~~~~~~~~~~~~~~~~~~~~~~~~~~~~~~~~~~~~~~~~~~~~~~~~~~~~~~
C  12Cm 1          618.000    1.520      1.520  |Methyl carbon to ethyl carbon
H  0 Cm 1          655.000    1.100      1.100  |Hydrogen to methyl carbon
C  12N+ 1          739.000    1.472      1.472  |Carbon to imidazolium nitrogen
```

Parameter	Units	Variable Type
Label_ij	None	Character*10
K_{Bond}	kcal/mol·Å^2	Double precision
R_0	Å	Double precision
R_0 *(constrained)*	Å	Double precision

The bond label, *Label_ij*, is a non-control text field which serves to identify the species of atoms involved in the referenced bond.

The bond parameters actually used within the simulation calculation begin with K_{Bond}, which represents the spring constant for the harmonic oscillator model, while R_0 represents the associated average bond length.

If the SHAKE algorithm is used to rigidly constrain the bonds, then R_0 *(constrained)* is utilized for the rigid bond length instead.

Any additional data beyond the value for *R0 (constrained)* is discarded and can therefore be used for additional notes about the defined bonds, as long as at least one space is present after the numerical value for *R0 (constrained)*.

Section 4

Bending Potentials

The bend energy between any three contiguous atoms employs a harmonic potential about the bend rest angle:

$$U_{Bend}\left(\theta_{\alpha\beta\gamma}\right)=\frac{1}{2}k_{\alpha\beta\gamma}^{Bend}\left(\theta_{\alpha\beta\gamma}-\theta_{\alpha\beta\gamma}^{0}\right)^{2}$$

The first line of the bend parameterization subsection dictates the number of unique bending potentials (T_{Bend}) defined within the system:

```
*                                                           Bends
    23
```

The remaining T_{Bend} lines in the bend potential subsection describe the individual bending interactions in ascending order, indexed from 1 to T_{Bend}. Each line consists of a (informative only) label and two parameters providing the bend energy constant ($k_{\alpha\beta\gamma}^{Bend}$) as well as the nominal bend angle (θ^0). Mapping of atoms to a specific bend types is accomplished in the `connectivity.dat` file.

```
*~~~~~~~~~~~~~~~~~~~~~~~~~~~~~~~~~~~~~~~~~~~~~~~~~~~~~~~~~~~~~~~~~~~~~~~~~~~~~~~~~~~~
* alpha_beta_gamma    K_Bend     Theta0     Linear
*~~~~~~~~~~~~~~~~~~~~~~~~~~~~~~~~~~~~~~~~~~~~~~~~~~~~~~~~~~~~~~~~~~~~~~~~~~~~~~~~~~~~
H  1 Cm 0 C  0        86.00      110.500    F     | from C4,C5 alkanes
H  0 Cm 1 H  0        77.00      107.700    F     | terminal methyl group
```

Parameter	Units	Variable Type
Alpha_beta_gamma	None	Character*15
K_{Bend}	kcal/mol·rad^2	Double precision
Theta0 (θ_0)	degrees	Double precision

The label *alphabetagamma* is a non-control label which serves to identify the species involved in the bending potential.

The bending potential parameters are comprised of K_{Bend} which specifies energy constant for the harmonic bending potential and θ_0 which specifies the reference angle for a bend containing atoms of species α, β, and γ in that order. The *Linear* option is currently not used for calculation. Any extra data after θ_0 is therefore discarded, and can therefore be used to provide additional notes about the bends defined.

Section 5

Torsional Parameters

The torsional energy between any four contiguously bonded atoms is specified by:

$$U_{Tors}\left(\phi_{\alpha\beta\gamma\delta}\right)=\frac{1}{2}\sum_{n} k^{Tors}_{\alpha\beta\gamma\delta}(n)\left[1-\cos\left(n\phi_{\alpha\beta\gamma\delta}\right)\right]$$

The first line of the torsional energy subsection of `ff.dat` provides the number of unique torsional interactions ($T_{Torsion}$) within the system:

```
*                                 Torsions
   21
```

The remaining $T_{Torsion}$ lines in the torsional energy subsection describe the individual torsional potentials in ascending order, indexed from 1 to $T_{Torsion}$. Each line consists of an informative label indicating the identity of the four species involved in the torsional paramaterization, followed by the total number of terms in the torsional expansion, and the value of $k^{Tors}_{\alpha\beta\gamma\delta}$ for each of these expansion terms. The maximum number of terms possible in the expansion is set at compilation time via the `params.h` file. Mapping of specific atoms to a torsional interaction type is accomplished via the `connectivity.dat` file.

```
* alpha_beta_gamma_delta  nprms  K0    K1    K2    K3    K4...
*~~~~~~~~~~~~~~~~~~~~~~~~~~~~~~~~~~~~~~~~~~~~~~~~~~~~~~~~~~~~~~~~~~~~~~~~~~~~~~
H  0 Cm 0 C  12N+ 1      4      0.00  0.00  0.00  -0.30  |Imidazolium H-C-C-C
H  0 Cm 0 C  0 H  0      4      0.00  0.00  0.00  -0.30
Cm 0 C  12N+ 1 Cc 0      3      0.00  0.00  0.43         |Imid+. Cm-C-N+-C
```

Parameter	Units	Variable Type
alpha_beta_gamma_delta	None	Character*20
nprms (n)	None	Integer
K0, K1, K2, K3, …, K_n	kcal/mol	Double precision

The label *alpha_beta_gamma_delta* is a non-control label which identifies the species of the four atoms for which the torsional parameters provided apply. The definition of the torsional energy term begins with *nprms* which is the number of potential energy terms utilized to build the complete torsional energy curve. Following this are *n* double precision values representing the appropriate force constants for the potential energy expansion, with at least a single space between each constant. Any extra data beyond the last force constant is disgarded, and can therefore be used for further notes about the defined torsions.

Section 6
Planar Deformations

The energy term utilized for planar deformations is:

$$U^{Deform}\left(\phi_{\alpha\beta\gamma\delta}\right)=\frac{K^{Deform}_{\alpha\beta\gamma\delta}}{2}\left(\phi_{\alpha\beta\gamma\delta}\right)^2$$

For this energy term, *j* represents the out-of-plane atom, and the only necessary forcefield parameter is the energy constant, $K_{\alpha\beta\gamma\delta}^{Deform}$

As in all previous subsections, the first line of this subsection denotes the number of unique out-of-plane interactions (T_{Deform}):

```
*                                    Out-of-plane deformations
    4
```

The next N_{Deform} lines provide requisite labels and the necessary energy constant for each unique interaction type, indexed from 1 to T_{Deform}:

```
* alpha_beta_gamma_delta    K_abgd
*~~~~~~~~~~~~~~~~~~~~~~~~~~~~~~~~~~~~~~~~~~~~~~~~~~~~~~~~~~~~~~~~~~~~~~~~~~~~~~~~~~
~
*
Cc 8 N+ 1 Cc 9 C  12        65.0
N+ 1 Cc 8 Cc 8 H  2         57.0
```

Parameter	Units	Variable Type
alpha_beta_gamma_delta	None	Character*20
K_abgd ($K_{\alpha\beta\gamma\delta}$)	kcal/mol·rad^2	Double precision

The label *alpha_beta_gamma_delta* is a non-control label which identifies the species which must be constrained in a planar fashion. The force constant, $K_{\alpha\beta\gamma\delta}$ determines the stiffness of the constraint that attempts to maintain planarity of the molecule. *The force constant $K_{\alpha\beta\gamma\delta}$ should always be positive. A value less than 1.0e-5 is used to indicate a planar constraint on the molecule, and the deformation will be forced to planarity.* Any extra data beyond the force constant is ignored.

Section 7

Lone Pair Definition

The final subsection of the `ff.dat` file contains the necessary details to define the off-atom force centers also called lone pair dummy atoms. For specific systems (e.g. water) the geometric requirements to obtain proper orientation due to local electronic interactions (e.g. hydrogen bonding) is not sufficiently accounted for by simple charge or dipole moments located at the center of the atom in question. For this reason, Lucretius™ implements the ability to explicitly describe lone pairs in one of three geometries. These lone pairs are treated as dummy off-atom force centers with rigidly constrained geometries. Since the dummy atoms are rigidly constrained based upon three atoms that define its basis, the resultant forces from non-bonded interactions are transferred to the host atoms (basis of three atoms) used to form the lone pair geometry.

There are three types of lone pair geometries currently implemented in Lucretius. Each type is represented by three values representing the geometric specification of the lone pair location:

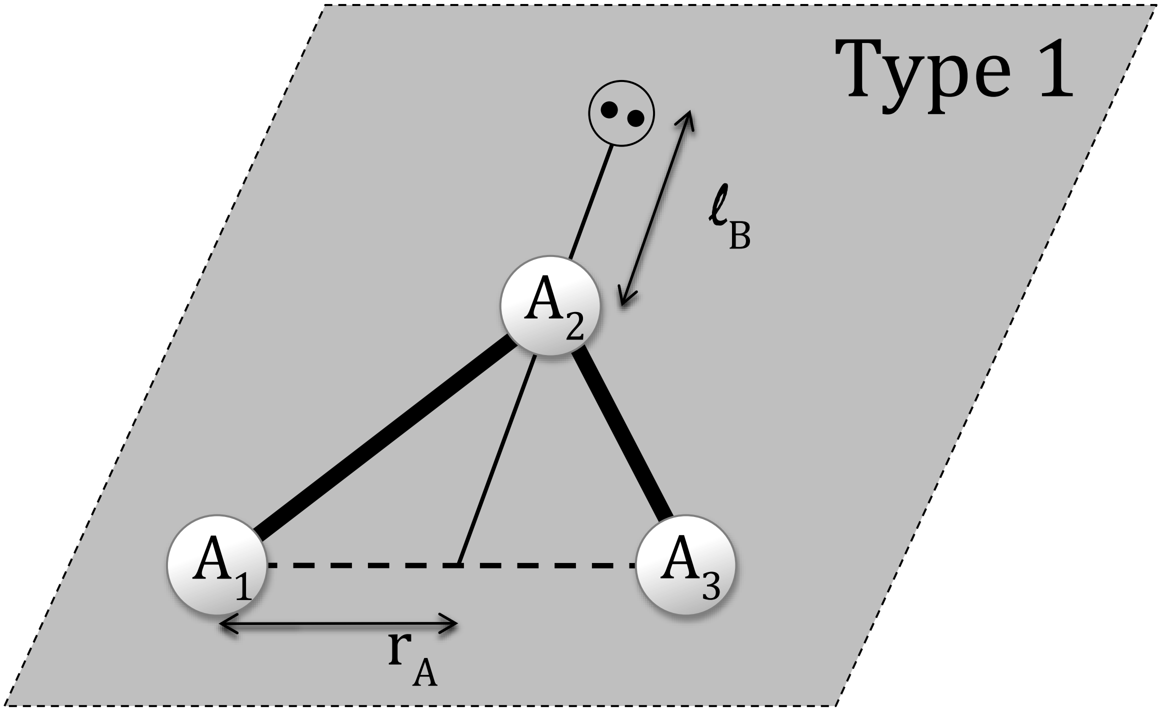

Type 1 lone pairs represent the lone pair with a single force center parallel to the bisector of angle A1-A2-A3. For such lone pairs, the length ℓ_B represents the distance between the atom to which the lone pair is attached and the center of the dummy force center representing the lone pair.

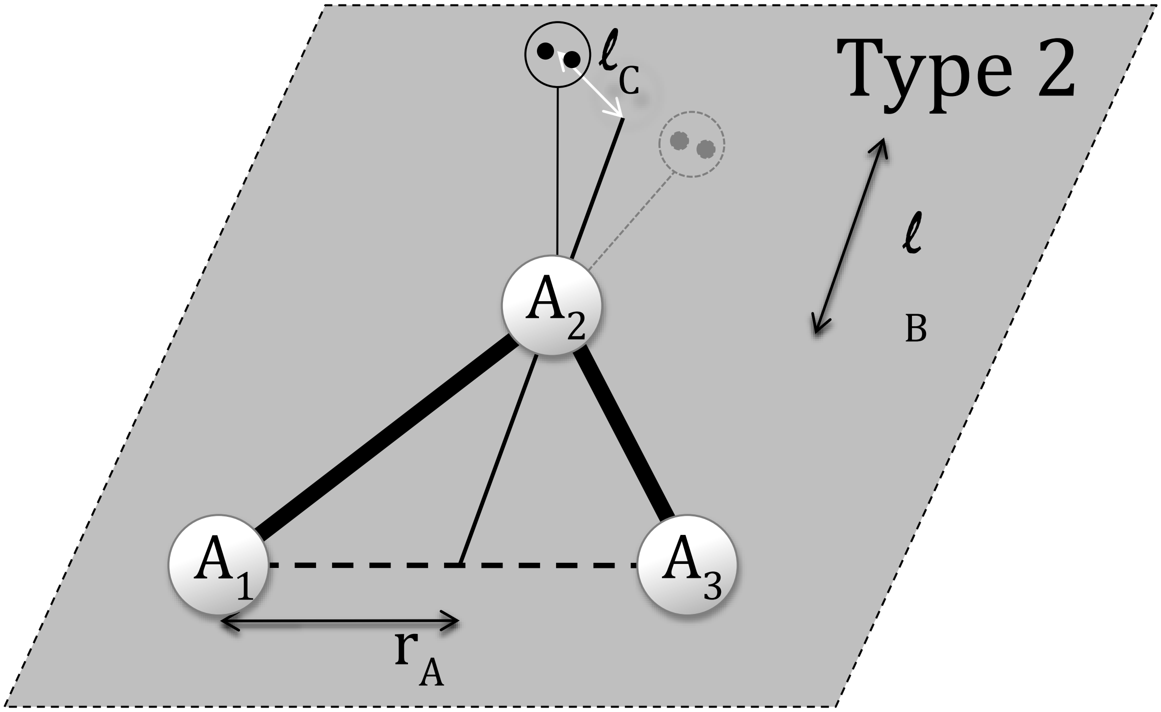

Type 2 lone pairs have two force centers attached to the central atom, extending out of the plane of the bend A1-A2-A3. Here, the distance from the central angle atom to the line which connects the center of the two dummy force centers is ℓ_B, while the distance from the plane of the atom to

the force centers, perpendicular to the plane is ℓ_C. The overall distance from the atom to the force centers is therefore $\sqrt{\ell_B^2 + \ell_C^2}$.

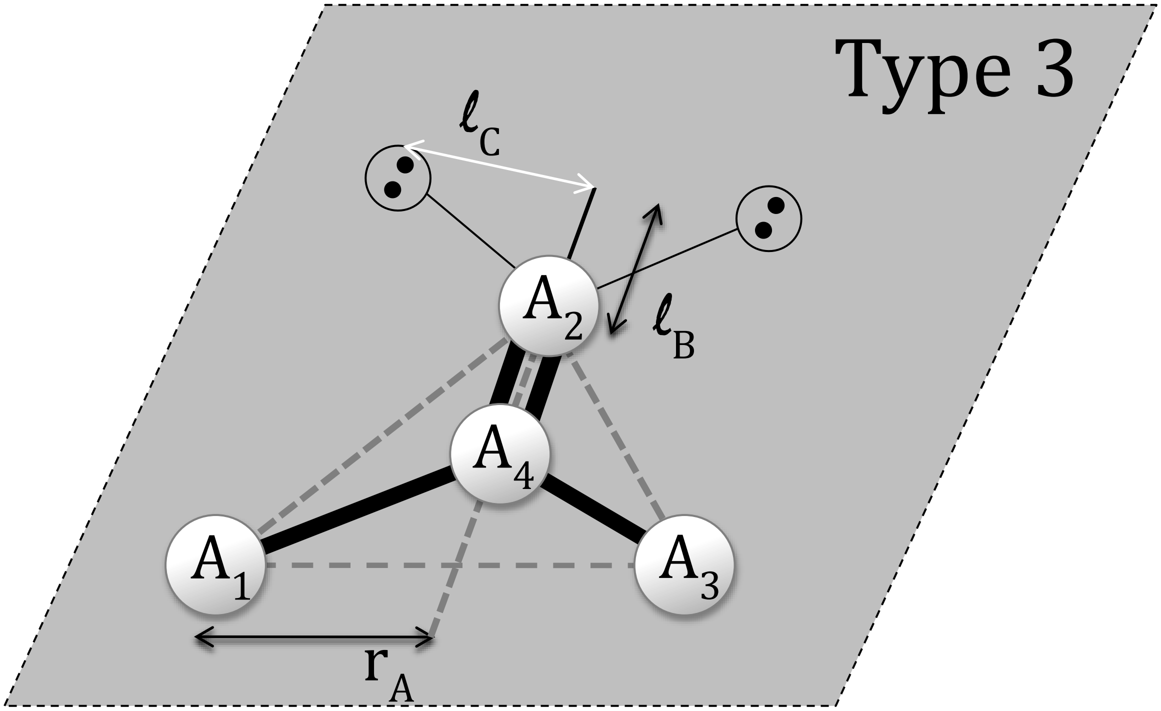

Type 3 lone pairs have two force centers attached to the central atom, extending in the plane of the bend A1-A2-A3. Here, the distance from the central angle atom to the line which connects the center of the two dummy force centers is ℓ_B, while the distance from the force center to the imaginary line formed by the bisector of angle A1-A2-A3 is ℓ_C. The overall distance from the atom to the force centers is again $\sqrt{\ell_B^2 + \ell_C^2}$. ***Please note that type 3 lone pairs are currently still experimental.***

In the current version of MD code dummy atoms r_A is always a half of r(A1-A3).

The lone pair subsection of the `ff.dat` file begins with the number of uniquely specified lone pair geometries (T_{LP}):

```
*                        Lone Pair definitions
   2
```

The remaining T_{LP} lines provide labels, the relevant charge type, and the necessary parameters to define the lone pairs in the system, indexed from 1 to T_{LP}:

```
* Bend Atoms       Charge Index  r_A    l_B        l_C       Type  A1  A2  A3
*~~~~~~~~~~~~~~~~~~~~~~~~~~~~~~~~~~~~~~~~~~~~~~~~~~~~~~~~~~~~~~~~~~~~~~~~~~~~~~
H  1 O  1 H  1     6             0.50   -0.23808  0.00000   1     3   4   3
Cc 1 O  2 Cc 1     7             0.50   0.409060  0.505140  2     6   5   6
```

<u>Parameter</u> <u>Units</u> <u>Variable Type</u>

Bend Atoms	None	Character*20
Charge Index	None	Integer
r_A	None	Double precision, must be 0.5
ℓ_B	Å	Double precision
ℓ_C	Å	Double precision
Type	None	Integer
A1	None	Integer
A2	None	Integer
A3	None	Integer

The label *Bend Atoms* is a non-control variable which identifies the species forming the bend upon which the lone pairs are rigidly fixed.

The *charge index* is an integer which contains the numeric index of the atom type to be considered a lone pair. This number is the index of the defined atom type which is used to represent the lone pair, where the index is derived by counting all previous charge combinations. For example, in the following snippet of a `ff.dat` file, the bolded, underlined numbers on the left represent the charge index of the related atomic species. Note that if a repulsion-dispersion type does not have explicit charges and polarizability, both quantities are assumed to be zero. In such a case, the affected atom *still counts as a charge species* (as in the first labeled charge species below):

```
      *~~~~~~~~~~~~~~~~~~~~~~~~~~~~~~~~~~~~~~~~~~~~~~~~~~~~~~~~~~~~~~~~~~~~~~~~~~~~
      * Label    A           B          C          D     Mass    Notes
      *   Charge      Induced polarizability
      *~~~~~~~~~~~~~~~~~~~~~~~~~~~~~~~~~~~~~~~~~~~~~~~~~~~~~~~~~~~~~~~~~~~~~~~~~~~~
1.    C          97431.00   3.62220    519.87     0.00  12.011  exp-6|6. C
      H          5352.30    4.36460    22.59      0.00  1.008    exp-6|1. H
2.         0.0576     0.3500     |1, H*-C Bonded to  C   8,
3.         0.1169     0.3500     |1, H*-C Bonded to  Cm  2,
      Lp         0.00       0.00000    0.00       0.00  0.000   lj126|Lone pr
4.         -1.1074    0.0000     |6, force center in SWM4 H2O Bonded to O
5.         -0.2200    0.0000     |1, ether group Bonded to O,
```

The next three parameters are used to fix the location of the lone pair relative to the molecular geometry of the atoms of the related bend:

- r_A – Relative proportion of the vector from one terminal atom (A1) to the other (A3) along which the line intersecting the central atom of the bend (A2) is drawn. This line, in turn, serves as the direction in which the length ℓ_B is extended. For current implementations, this value should be set to 0.5 to approximate the bisection of the A1-A2-A3 angle.
- ℓ_B – The (projected) distance along the line (plane) containing the bisector from the central atom of the affected bend. To locate the lone pair exterior to the bend, as in dimethoxyethane, ℓ_b should be positive. To locate the lone pair interior to the bend, as in TIP4P water, ℓ_b should be negative.
- ℓ_C – In a type 1 lone pair, ℓ_C should be zero. For a type 2 lone pair, ℓ_C represents the perpendicular distance from the plane containing the atoms A1-A2-A3 to the dummy force centers (one at - ℓ_C the other at ℓ_C). For a type 3 lone pair, ℓ_C

represents the perpendicular distance from the bisector of A1-A2-A3 to the dummy force centers (one at - ℓ_C the other at ℓ_C).

The *LP Type* specifies whether the lone pairs are represented by a dummy atom with a single lobe in the plane of the bend (*type 1*), by two dummy atoms which form a tetrahedrally coordinated structure with the related central bend atom (*type 2*), or by two dummy atoms which form a planar structure with the related central bend atoms (*type 3*).

Finally, the A1, A2, and A3 parameters correspond to the *charge indices* (see above) for the atom types of the A1, A2, and A3 atom positions in the lone pair geometry. This allows for both error checking to ensure that lone pairs are placed on the proper atoms, as well as the unambiguous specification of location in type 3 lone pairs, where the construction geometry differs from the bonding connectivity.

coords.inp/coords.out

The `coords.inp` and `coords.out` file both have the same format. These files represent a complete summary of the instantaneous positions and velocities of all the atoms in the system in a plain ASCII format, as well as containing information about the system cell size and the state of any extended lagrangian variables within the system. Since both files have identical formats, we will refer to them as simply "coordinate files".

The coordinate files have three major portions:

1. An informational header which may store a text description of the system simulated
2. A list of positions and velocities, one set of each for every force center in the system
3. An extended system which provides the definition of the simulation cell at a minimum, and may also provide extended coordinates for thermostat and barostat variables.

Section 1

The coordinate files begin with a header comprised solely of an arbitrary number of comment lines (lines whose first character is an asterisk) which are generally used to provide useful information about the system.

Section 2

Following the introductory header is a section comprised of N_{fc} blocks (where N_{fc} is the number of individually defined simulation force centers), with each block comprised of two lines with the following format:

```
*     X               Y               Z          Atom  Charge
*          V_X             V_Y             V_Z
*---------------------------------------------------------------------------
*
     22.538988       25.630641       10.683374  Cm    2
          -24.740393      -235.265295     -106.378173
     22.583869       24.214686       11.234227  C     1
          -157.029719     -392.865083     -497.809885
     22.744302       24.144902       12.695788  N+    1
          275.429794      -242.863373     -537.171476
```

Parameter	Units	Variable Type
X	Å	Double precision
Y	Å	Double precision
Z	Å	Double precision
Atom Type (Atom)	None	Character*3
Charge Subtype (Charge)	None	Integer
V_X	m/s	Double precision
V_Y	m/s	Double precision

V_Z	m/s	Double precision

Line 1

X, Y, and *Z* provide the Cartesian coordinates which describe the location of the relevant force center. The valid range of these values is from 0.0 to B_X, B_Y, and B_Z, respectively, where B_i is the length of the simulation box in the *i* direction.

After the Cartesian coordinates, the *atom type* label associated with the proper species in the `ff.dat` file is provided, as well as the relevant charge subtype within that species. Valid values for charge subtype are from 1 to $N_{Q\text{-}Label}$ where $N_{Q\text{-}Label}$ is the total number of charge subtypes for the species of type *label*.

Line 2

V_X, V_Y, and V_Z provide the *X*, *Y*, and *Z* components of the velocity of the referenced atom.

Section 3

At the end of the coordinate files is an additional section which contains the necessary information to specify the simulation cell and any extended variables necessary for simulation in ensembles other than the microcanonical.

```
*-------------------------------------------------------------------------
* Box (Angstroms)
35.1971066497367
*     Xlogs (Thermostat position)
           -2.18539477964628
*     Vlogs (Thermostat velocity)
           1.494671827159197E-004
*     Glogs (Thermostat force)
           2.931502988086876E-006
*     Xlogv (Barostat position)
           -5.366430103976744E-004
*     Vlogv (Barostat velocity)
           -6.873090614127863E-006
*     Glogv (Barostat force)
           7.293176555254246E-008
```

Parameter	Units	Variable Type
Box (L_{Box})	Å	Double precision
Xlogs	Å	Double precision
Vlogs	m/s	Double precision
Glogs		Double precision
Xlogv	Å	Double precision
Vlogv	m/s	Double precision
Glogv		Double precision

The size of the box is given in the section marked *Box*. If the system is cubic, a single value is listed, while an orthorhombic system will have three values representing the box length in the *X*, *Y*, and *Z* directions respectively.

Xlogs, Vlogs, and *Glogs* represent the variables necessary to track the thermostat evolution in an NVT or NPT simulation. Each section will have N_{NHC} values *(see* `mdrun.params`*)* representing the thermostat "position", "velocity", and "force" of the indexed Nosé-Hoover chain temperature coupling to the virtual "heat bath". At the beginning of a run, or after equilibration, it is generally recommended to set all of these variables to 0.0 in order to "erase" the thermostat history before generating data for a production run. Otherwise, these values are generally not modified except through the simulation itself.

Xlogv, Vlogv, and *Glogv* represent the variables necessary to track the barostat evolution in an NPT simulation. Here, each section has either one (cubic) or three (orthorhombic) values which house the associated "position", "velocity", and "force" of the Nosé-Anderson barostat mass which is used to control the pressure of the system. Like the thermostat variables, it is recommended to set all the barostat associated quantities to 0.0 at the beginning of a production set of simulation runs and to otherwise not modify these values outside of the normal process of running a simulation in the NPT ensemble.

connectivity.dat

The `connectivity.dat` file provides the molecular connectivity information necessary to completely specify the atoms, bonds, bends, torsions and deformations of each molecular species within the system. The `connectivity.dat` file is comprised of five separate sections: The first section dictates the number of molecular and atomic units within the system. The second section provides the number of atoms, bonds, bends, torsions and deformations per unit. The third section provides a list of all the bonds, which are mapped to molecules via the information in section two and the table of force centers provided through `coords.inp`. The fourth, fifth, and sixth sections provide a list of all the bends, torsions, and deformations which are mapped in the same manner as the bonds.

For ease of reference, we will collectively refer to either a molecule or an isolated single atom/ion as a *unit*, while the individual atoms/lone pairs which comprise the molecule or atom/ion will be referred to as a *force center*.

<u>Section 1</u>
Unit declarations

The first section provides the number of molecules (N_{Mol}) and single atoms/ions (N_{Single}). Each unit must be defined as one or the other.

```
*~~~~~~~~~~~~~~~~~~~~~~~~~~~~~~~~~~~~~~~~~~~~~~~~~~~~~~~~~~~~~~~~~~~~~~~~~~~~
*     Number of Molecules   Single atoms/ions
*~~~~~~~~~~~~~~~~~~~~~~~~~~~~~~~~~~~~~~~~~~~~~~~~~~~~~~~~~~~~~~~~~~~~~~~~~~~~
      4                     1
```

Parameter	Units	Variable Type
Number of Molecules (N_{Mol})	None	Integer
Single Atoms/Ions (N_{Single})	None	Integer

<u>Section 2</u>
Unit interaction list

The second section provides $N_{Mol}+N_{Single}$ lines (one for each unit). The ith line in the section lists the number of force centers, bonds, bends, torsions and deformations for the corresponding i^{th} unit in the system.

```
*~~~~~~~~~~~~~~~~~~~~~~~~~~~~~~~~~~~~~~~~~~~~~~~~~~~~~~~~~~~~~~~~~~~~~~~~~~~~
*                         Chain configuration
*
*     Atoms      Bonds      Bends      Torsions   Deformations
*~~~~~~~~~~~~~~~~~~~~~~~~~~~~~~~~~~~~~~~~~~~~~~~~~~~~~~~~~~~~~~~~~~~~~~~~~~~~
      19         19         33         41         5
      19         19         33         41         5
      19         19         33         41         5
      19         19         33         41         5
      1          0          0          0          0
```

Parameter	Units	Variable Type
Atoms (N^i_{FC})	None	Integer
Bonds (N^i_{Bond})	None	Integer
Bends (N^i_{Bend})	None	Integer
Torsions (N^i_{Tors})	None	Integer
Deformations (N^i_{Deform})	None	Integer

The *Atoms* (N^i_{FC}) value determines how many force centers are in the current (i^{th}) molecule, with the force centers assigned in sequential order from the list generated from the `coords.inp` file. Thus, in the preceding example, the `coords.inp` file would be expected to contain 77 unique force center descriptions, with the information for force centers 1-19 ($N^1_{FC} = 19$) representing the coordinates for the members of molecule 1, 20-38 for molecule 2 ($N^2_{FC} = 19$), and so on, up to coordinate number 77 ($N^5_{FC} = 1$)representing the data for the sole single atom/ion in the system.

The *Bonds* (N^i_{Bond}) value determines how many bonds are in the current (i^{th}) unit, with the bonds assigned from the list of bonds in *Section 3* of `connectivity.dat`. The bonds are assigned sequentially, with the first N^1_{Bond} bonds assigned to molecule 1, the next N^2_{Bond} bonds assigned to molecule 2, and so on. For single force center units, even though $N^x_{Bond} = 0$, *Section 3* is required to have a single line which explicitly demonstrates the lack of bonds with the referenced force center. Thus, the total length of the bond list is $\sum_i N^i_{Bond} + N_{Single}$.

The *Bends* (N^i_{Bend}) value determines how many bends are in the current (i^{th}) unit, with the bends assigned from the list of bends in *Section 4* of `connectivity.dat`. The bends are assigned sequentially, with the first N^1_{Bend} bends assigned to molecule 1, the next N^2_{Bend} bends assigned to molecule 2, and so on.

The *Torsions* (N^i_{Tors}) value determines how many torsions are in the current (i^{th}) unit, with the torsions assigned from the list of torsions in *Section 5* of `connectivity.dat`. The torsions are assigned sequentially, with the first N^1_{Tors} torsions assigned to molecule 1, the next N^2_{Tors} torsions assigned to molecule 2, and so on.

The *Deformations* (N^i_{Deform}) value determines how many deformations are in the current (i^{th}) unit, with the deformations assigned from the list of deformations in *Section 6* of `connectivity.dat`. The deformations are assigned sequentially, with the first N^1_{Deform} deformations assigned to molecule 1, the next N^2_{Deform} deformations assigned to molecule 2, and so on.

<u>Section 3</u>

List of bonds

The third section provides the bond list, which is used to determine the bonds present in the molecules as well as to explicitly denote non-bonded single force center units. The bond list should contain $N_{Bond}^{Total} = \sum_i N_{Bond}^i + N_{Single}$ lines. An example bond list follows:

```
*                   Bonds
*
*       FC_i        FC_j        Bond Type
*~~~~~~~~~~~~~~~~~~~~~~~~~~~~~~~~~~~~~~~~~~~~~~~~~~~~~~~~~~~~~~~~~~~~~~~~~~~~~~~
        1           2           1
        1           9           2
        1           10          2
        1           11          2
        2           3           3
        …
        77
```

Parameter	Units	Variable Type
FC_i	None	Integer
FC_j	None	Integer
Bond Type	None	Integer

The values of FC_i and FC_j specify the force centers (*i* and *j*, respectively) which comprise the specified bond.

The *Bond Type* specifies the relevant parameters for the bond energy/force calculation by referencing the relative position in the bond type subsection of the `ff.dat` file. (*Section 3* of `ff.dat`)

The final example bond above provides an example of the required statement for a single force center unit. Here, only FC_i is defined, with the rest of the line intentionally left blank to demonstrate the unbonded nature of the atom.

Section 4
List of bends

The fourth section provides the bend list, which is used along with the information from *Section 2* to determine which bend interactions are present within which units. The bend list should contain $N_{Bend}^{Total} = \sum_i N_{Bend}^i$ lines. An example bend list follows:

```
*                   Bends
*
*       FC_i        FC_j        FC_k        Bend Type
*~~~~~~~~~~~~~~~~~~~~~~~~~~~~~~~~~~~~~~~~~~~~~~~~~~~~~~~~~~~~~~~~~~~~~~~~~~~~~~~
        9           1           2           1
        10          1           2           1
        11          1           2           1
        10          1           9           2
…
```

Parameter	Units	Variable Type
FC_i	None	Integer
FC_j	None	Integer
FC_k	None	Integer
Bend Type	None	Integer

The values of FC_i, FC_j, and FC_k specify the force centers (*i*, *j*, and *k* respectively) that comprise the specified bend. Here, *j* should represent the force center interior to the bend.

The *Bend Type* specifies the relevant parameters for the bend energy/force calculation constants by referencing the relative position in the bend type subsection of the ***ff.dat*** file. (*Section 4* of ***ff.dat***)

Section 5
List of torsions

The fifth section provides the torsion list, which is used along with the information from *Section 2* to determine which torsional interactions are present within which units. The torsion list should contain $N_{Torsion}^{Total} = \sum_i N_{Torsion}^i$ lines. An example torsion list follows:

```
*          Torsions (Dihedrals)
*
*     FC_i       FC_j       FC_k       FC_l       Torsion Type
*~~~~~~~~~~~~~~~~~~~~~~~~~~~~~~~~~~~~~~~~~~~~~~~~~~~~~~~~~~~~~~~~~~~~~~~~
      9          1          2          3          1
      9          1          2          12         2
      9          1          2          13         2
      10         1          2          3          1
      10         1          2          12         2
...
```

Parameter	Units	Variable Type
FC_i	None	Integer
FC_j	None	Integer
FC_k	None	Integer
FC_l	None	Integer
Torsion Type	None	Integer

The values of FC_i, FC_j, FC_k and FC_l specify the force centers (*i, j, k* and *l* respectively) that comprise the specified torsional angle. Here, *j* should represent the force center interior to the bend *ijk* and *k* the force center interior to the bend *jkl*.

The *Torsion Type* specifies the relevant parameters for the torsional energy/force calculation constants by referencing the relative position in the torsion type subsection of the ***ff.dat*** file. (*Section 5* of ***ff.dat***)

Section 6

List of deformations

The sixth and final section provides the deformation list, which is used along with the information from *Section 2* to determine which force centers within which units have interactions which enforce planarity of a quartet of bonded atoms. The deformation list should contain $N_{Deform}^{Total} = \sum_i N_{Deform}^i$ lines. An example deformation list follows:

```
*     Out-out-plane deformations (Improper Torsions)
*     FC_i        FC_j        FC_k        FC_l        Deform Type      Inside
*~~~~~~~~~~~~~~~~~~~~~~~~~~~~~~~~~~~~~~~~~~~~~~~~~~~~~~~~~~~~~~~~~~~~~~~~~~~~
      5           3           4           2           1                1
      6           4           3           14          2                1
      7           5           3           15          3                1
      7           6           4           16          2                1
```

Parameter	Units	Variable Type
FC_i	None	Integer
FC_j	None	Integer
FC_k	None	Integer
FC_l	None	Integer
Deform Type	None	Integer
Inside	None	Integer

The values of FC_i, FC_j, FC_k and FC_l specify the force centers (*i, j, k* and *l* respectively) that comprise the specified planar force center quartet. Here, *j* should represent the central force center and should be bonded to *i,k,* and *l*.

The *Deform Type* specifies the relevant parameters for the deformation energy/force calculation constants by referencing the relative position in the deform type subsection of the ***ff.dat*** file. (*Section 6* of ***ff.dat***)

The *Inside* parameter can take the value of either **-1** or **1** only, and is used as a flag to determine whetherthe force center represented by FC_l is interior to angle *ijk*. If *Inside* is -1 *l* is interior to angle *ijk*, while a value of 1 means *l* is exterior to angle *ijk*.

params.h

The params.h file is part of the compile-time control file set for the Lucretius™ simulation package. Multiple values within the params.h file must be properly set in order for Lucretius™ to function properly. This section provides a list of the parameters within params.h and discusses appropriate values for these parameters. These parameters may be defined in any order (although some parameters depend on previously defined parameters and may thus be kept in the specified order for the definition to make sense.

```
C Parameters for unit and force center list
      integer maxnch             !Maximum number of molecules + atoms
         parameter (maxnch= 250)
      integer maxat              !Maximum number of force centers
         parameter (maxat= 3500)
C
C Parameters for connectivity lists
      integer maxbonds           !Maximum number of bonds in system
         parameter (maxbonds= 3375)
      integer maxbends           !Maximum number of bends in system
         parameter (maxbends= 5750)
      integer maxtorts           !Maximum number of torsions in system
         parameter (maxtorts= 5875)
      integer maxdeforms         !Maximum number of deformations in system
         parameter (maxdeforms= 625)
C
C Parameters for force field
      integer maxtypes           !Max number of repulsion-dispersion types
         parameter (maxtypes= 9)
      integer maxcharges         !Max number of charge types
         parameter (maxcharges= 16)
      integer maxdummy           !Max number of dummy (lone pair) atoms
         parameter (maxdummy= 0)
      integer maxbtypes          !Max of types(bonds,bends,torts)
         parameter (maxbtypes= 23)
      integer maxfolds           !Max number of terms in torsional energy
         parameter (maxfolds = 9 )
C
C Parameters for neighbor list
      integer maxbox             !Maximum force centers per cell subbox
         parameter (maxbox = 25)
      integer maxnay             !Maximum neighbors per force center
         parameter (maxnay = 999)
      integer maxdim             !Maximum box subdivisions per axis
         parameter (maxdim = 25)
      integer maxdim3            !Total number of box subdivisions
         parameter (maxdim3 = maxdim*maxdim*maxdim)
C
C Polarization related parameters
      real*8 a_thole             !Dipole-dipole damping constant
         parameter (a_thole=0.2d0)
C
C Misc additional parameters as indicated (advanced)
      integer maxpoints          !Max points in interaction splines
         parameter (maxpoints = 501)
      integer maxprop            !Max number of system averaged properties
         parameter (maxprop = 30)
      integer maxnnb             !Max number of non-bonded interactions
```

```
   parameter (maxnnb = maxcharges*(maxcharges+1)/2)
integer kmax               !Max k vectors in one direction
   parameter (kmax = 6)
```

Section 1

The first two parameters control the length of the lists which hold the individual data about each force center as well as the overarching data about each unit (number of bonds, bends, torsions, etc):

maxnch — The maximum number of units (molecules and atoms/ions) within the current simulation. ($N_{Mol} + N_{Single} \le maxnch$, see ***connectivity.dat***)

maxat — The maximum number of force centers within the current simulation. ($N_{FC} \le maxat$, see ***coords.inp***)

Section 2

The next four parameters hold the size data for the bond, bend, torsion, and out-of-plane deformation lists:

maxbonds — The maximum number of individual bond definitions within the simulation. ($N_{Bond}^{Total} \le maxbonds$, see ***connectivity.dat**, Section3*)

maxbends — The maximum number of individual bend definitions within the simulation. ($N_{Bend}^{Total} \le maxbends$, see ***connectivity.dat**, Section 4*)

maxtorts — The maximum number of individual torsion definitions within the simulation. ($N_{Torsion}^{Total} \le maxtorts$, see ***connectivity.dat**, Section 5*)

maxdeforms — The maximum number of out-of-plane deformations within the simulation. ($N_{Deform}^{Total} \le maxdeforms$, see ***connectivity.dat**, Section 6*)

Section 3

The next three parameters hold necessary information for the size of data structures dictated by the employed forcefield:

maxtypes — The maximum number of repulsion-dispersion interaction types. This value does not take into account differing charge/polarizability species. ($T_{RD} \le maxtypes$, see ***ff.dat***, *Sections 1 and 2*)

maxcharges — The maximum number of different non-bonded interaction types *including charge/polarizability* species. (see ***ff.dat***, *Sections 2 and 7*)

maxdummy — The maximum number of "dummy" force centers utilized in the simulation to represent lone pairs for the system. There will be one dummy force center for every lone pair of type 1 (${}_1N_{LP}$), and two dummy force centers for every lone pair of type 2 (${}_2N_{LP}$). (

$_1N_{LP} + 2_2N_{LP} \leq maxdummy$, see ***ff.dat***, *Section 7* for further details about lone pair representation)

maxbtypes — The maximum of the number of *types* of bonds, bends, torsions, or out-of-plane deformations found in the ***ff.dat*** file. ($maxbtypes = \max(T_{Bond}, T_{Bend}, T_{Torsion}, T_{Deform})$, see ***ff.dat**, Sections 3-6*)

maxfolds — The maximum number of individual torsional terms allowed in the torsional energy definitions. (see ***ff.dat***, *Section 5* for details on torsional energy term expressions)

Section 4

The next four values contain information related to the neighbor list implementation utilized by Lucretius™. Lucretius™ calculates the neighbor list only when necessary (see ***mdrun.params**, Line 2*), utilizing a cell-based approach to organizing the force centers to further accelerate neighbor list formation. The simulation cell is subdivided into many equal volume sub-cells, and the individual force centers are indexed into the appropriate sub-cell. The neighbor list calculation then proceeds through all force centers, checking only those nearby sub-cells that might fall within the total interaction distance placing those which are inside the total interaction distance into the appropriate neighbor list. The following variables determine the size, number, and length of the sub-cells as well as the length of the neighbor list:

maxbox — The maximum number of force centers which can be indexed per sub-cell.

maxnay — The maximum number of neighbors allowed in a neighbor list. If Lucretius™ attempts to add more atoms to the neighbor list than it can contain, the simulation will be terminated with an appropriate error message instructing the user to increase this number.

maxdim — The maximum number of sub-cells in any given direction within the unit cell. For example, a *maxdim* value of 10 will subdivide the overall system cell into 10 subcells along each principle Cartesian vector.

maxdim3 — $maxdim^3$ – The total number of sub-cells within the system. A quick estimate for sufficient sub-cell numbers is: $maxdim^3 \times maxbox \approx 1.2N_{FC}$, assuming homogeneous spatial distribution of the force centers. As the spatial distribution of the force center becomes less homogeneous, the prefactor on the right hand side of the preceding equation increases.

Section 5

This parameter is used to delocalize the dipole-dipole interaction in order to try to avoid a "polarization catastrophe"

a_{thole} — The damping constant for Thole screening of the dipole-dipole interaction.

Section 6

The remaining parameters in the ***params.h*** file control miscellaneous variables for the internal workings of Lucretius™ and should generally be left as received. There are occasions, however, where they will need to be changed for specific applications, and as such they are summarized below:

maxpoints	The maximum number of discrete points utilized in construction of the spline which is utilized to evaluate the real-space component of the non-bonded interactions.
maxprop	The maximum number of time-averaged properties which can be tracked by Lucretius as the simulation progresses.
maxnnb	An automatically generated quantity which sets the appropriate size for the interaction matrix used to track the various components of non-bonded interactions between atom types.

Binary output file specifications

There are four potential binary output files which can be generated via Lucretius™. Whether or not each file is created, as well as the interval of output if the file is created, is controlled via lines 5 and 6 of the ***mdrun.params*** file. Each file contains an arbitrary number of snapshots of the relevant data and any associated header information, and is output in a purely binary format from Lucretius™. These files are generally used as a compact method (compared to ASCII output) for saving data for which the desired analysis is too time consuming or otherwise unsuitable for being performed during the execution of the simulation. The four potential file types are:

Total Box Dipole and Charge Flux – filename: *fort.76*

This file is composed of an arbitrary number of two-line blocks. In each block, the first line contains a single value giving the time interval at which the dipole and flux are calculated (t_{Dipole}). The second line contains the total box dipole (in Åe) in the X, Y, and Z direction (D_X, D_Y, and D_Z respectively) as well as the portion of the total box dipole due to the dipole-dipole interactions (in Åe) in the X, Y, and Z direction (P_X, P_Y, and P_Z respectively). The variable template for the output block is:

Real*4
6 × Real*4

Instantaneous Coordinates – filename: *fort.77*

This file is composed of an arbitrary number of two-line blocks. In each block, the first line contains four values representing the total number of force centers (N_{FC}), the time interval at which instantaneous coordinates are output (t_{Coords}), the current snapshot index (S_i), and the size of the simulation cell (L_{Box}). The second line contains N_{FC} triplets comprising the X, Y, and Z coordinates of each force center, respectively. The variable template for the output block is:

Integer, Real*4, Integer, Real*8
N_{FC} × 3 × Real*4

Pressure Tensor – filename: *fort.78*

This file is composed of an arbitrary number of two-line blocks. In each block, the first line contains a single value representing the time interval at which the stress tensor is calculated (t_{Stress}). The second line contains six values comprising the values of the symmetrized pressure tensor: σ_{xx}, σ_{yy}, σ_{zz}, σ_{xy}, σ_{xy}, σ_{yz}. The variable template for the output block is:

Real*4
6 × Real*4

Instantaneous Velocities – filename: *fort.79*

This file is composed of an arbitrary number of two-line blocks. In each block, the first line contains two values, the total number of force centers (N_{FC}) and the time intervel at which instantaneous velocities are output (t_{Veloc}). The second line contains N_{FC} triplets comprising the X, Y, and Z components of the velocities (in m/s), respectively. The variable template for the output block is:

Integer, Real*4
$N_{FC} \times 3 \times$ Real*4

Trace file (fort.65)

The fort.65 file produces an ongoing trace of the average simulation quantities over the time window specified in *Line 4* of the ***mdrun.params*** file. The file format is straightforward, utilizing the following structure:

```
#-----------------------------------------------------------------------
--
#                          Running Averages
#
#     Time        Temp        Pressure    Box         Total E
      Hamiltonian
#     fs          K           Atm         Ang         Kcal/mol    Kcal/mol
#-----------------------------------------------------------------------
--
      150.00      294.53      323.637     35.2207     -20839.15   -20367.43
      300.00      293.17      208.734     35.2451     -20885.07   -20368.97
      450.00      300.07      -82.525     35.2871     -20796.82   -20370.32
      600.00      301.04      -275.374    35.3214     -20760.64   -20369.75
      750.00      295.31      -212.401    35.3315     -20807.94   -20369.89
      900.00      295.22      -2.149      35.3265     -20780.72   -20371.50
      1050.00     305.55      -48.291     35.3183     -20699.03   -20370.61
      1200.00     297.98      -43.759     35.3074     -20801.79   -20368.89
```

Here, the values presented represent the average value over the preceding time period, with the units as indicated in the header. While a variety of different properties may be calculated and output to the trace file depending on interest, there is currently no way to change the output values without requiring manipulation of the underlying simulation code and recompilation of the program. The values output are those which are generally of most interest:

Time – Current simulation time in femtoseconds.

Temp – Average temperature over the preceding time interval, in Kelvin.

Pressure – Average pressure over the preceding time interval, in Atmospheres.

Box – Average L_B over the preceding time interval, in Angstroms.

Total E – Average total energy over the preceding time interval, in kCal/mol.

Hamiltonian – Average Hamiltonian (including any extended contributions due to differing ensembles) over the preceding time interval, in kCal/mol.
